# The Hallucinogen Rating Scale: Updated Factor Structure in a Large, Multistudy Sample

**DOI:** 10.1016/j.bpsgos.2024.100436

**Published:** 2024-12-19

**Authors:** Abigail E. Calder, Clifford Qualls, Gregor Hasler, David Elmiger, Rick Strassman

**Affiliations:** a Molecular Psychiatry Laboratory, Faculty of Science and Medicine, University of Fribourg, Fribourg, Switzerland; b Department of Mathematics and Statistics, University of New Mexico, Albuquerque, New Mexico; c Freiburg Mental Health Network, Villarssur-Glâne, Switzerland; d Lake Lucerne Institute, Vitznau, Switzerland; e Department of Psychiatry, University of New Mexico School of Medicine, Albuquerque, New Mexico

**Keywords:** Factor analysis, Hallucinogen Rating Scale, Phenomenology, Psychedelics, Psychometrics, Questionnaires

## Abstract

**Background:**

The Hallucinogen Rating Scale (HRS) has been widely used to measure the subjective effects of psychedelics and other psychoactive substances. Its advantages include a basis in phenomenological interviews and clinical studies, straightforward items, and broad coverage of psychedelic effects. Previous studies have attempted to resolve its factor structure but were limited by small samples of participants who took only one substance.

**Methods:**

We obtained 991 HRS questionnaires from the authors of 18 publications involving 13 psychoactive substances. Exploratory factor analysis was used to analyze its factor structure, and mixed-effects analyses of variance were used to compare HRS scores between drugs.

**Results:**

The HRS resolved into 8 factors with good to excellent internal consistency and that intuitively map onto the effects of psychedelics. The factor model also showed good measures of fit that were superior to previous proposed models. Model factors were able to show dose responses for most drugs. Additionally, patterns of responses on the 8 factors significantly differentiated classic psychedelics, such as psilocybin and DMT, from other substance classes, including dissociatives such as ketamine and salvinorin A, empathogens such as MDMA, stimulants such as methylphenidate and amphetamine, and Δ^9^-tetrahydrocannabinol. The factor of meaningfulness also uniquely differentiated psychedelics from all other substances.

**Conclusions:**

These data show that the HRS is an intuitive and psychometrically sound tool for measuring the effects of psychedelic drugs, and it may also have utility for measuring the effects of other drugs and altered states of consciousness.

## Plain Language Summary

Calder *et al.* have updated the Hallucinogen Rating Scale, a popular instrument for assessing drug-induced altered states of consciousness. They retrieved data from 18 studies and 13 psychoactive substances, including psychedelics, dissociatives, empathogens, and others. Factor analysis revealed 8 intuitive and psychometrically sound factors that improve the questionnaire’s function. These factors can distinguish between doses, and different drug classes show specific patterns of effects. The factor “Meaningfulness” seems to be uniquely sensitive to the effects of psychedelics.

Psychedelics produce an extraordinary range of alterations in perception, emotion, and cognition, with specific effects strongly influenced by the individual and the environment in which they are taken, i.e., set and setting (1,2). Several drug classes with different mechanisms of action possess psychedelic properties, including classic psychedelics such as DMT, LSD, and psilocybin/psilocin; dissociatives such as ketamine and salvinorin A; and phenethylamines such as mescaline, 2C-B, and MDMA (3). After a several-decade lull in clinical research, psychedelics are once again gaining attention for their potential therapeutic applications, as well as their uses for personal growth, spirituality, and recreation (4).

Several questionnaires are used to quantify subjective psychedelic effects. The Hallucinogen Rating Scale (HRS) was developed in the 1990s based on reports of experiences with DMT (5). The HRS has been used in dozens of studies assessing the effects of psychedelics and other drugs, including psilocybin (6, 7, 8), ayahuasca (9, 10, 11), 2C-B (12), ketamine (13,14), MDMA (15,16), MDE (17), meta-chlorophenylpiperazine (mCPP) (18), salvinorin A (19), and new psychoactive substances (20). It has also been used to characterize non–drug-altered states of consciousness, such as near-death experiences (21). It has been translated into several languages, including Spanish (22), Portuguese (23), and German (17).

The 105-item HRS was originally based on detailed phenomenological interviews with experienced psychedelics users and then refined in subsequent studies of intravenous DMT (24,25). This led to straightforward item formulations assessing fundamental components of experience, and the authors avoided basing questions on spiritual, theological, or other higher-order interpretations of psychedelic effects. Items were originally grouped into 6 clinical clusters constructed by integrating a mental status examination model with Buddhist psychological deconstruction of ongoing mental experience, namely somaesthesia (physical/somatic effects), affect, perception, cognition, volition, and intensity (5).

After its initial development, 2 research groups investigated the HRS’s psychometric properties and factor structure in ceremonial ayahuasca users. They used a shortened HRS consisting of 71 items that had previously demonstrated a significant dose-response effect for DMT (22,26). The first study of 131 volunteers proposed a 2-factor solution based on principal component analysis (22). The second study involved 158 participants and proposed a 6-factor solution using exploratory factor analysis (EFA) (26).

However, studying single drugs in relatively homogeneous settings might have led to factor structures that reflect aspects of a particular setting in addition to drug effects. Furthermore, it is unlikely that previous factor analyses of the HRS included an adequate number of participants according to current recommendations. Sample sizes of between 5 and 10 participants per item, or alternatively at least 200 participants, have been recommended to obtain optimal results with EFA (27). More sophisticated estimates based on Monte Carlo simulations recommend at least 200 to 300 participants, especially when factor loadings and communalities are relatively low (27). Therefore, the factor structure of the full HRS would need to be evaluated in a sample of at least 200 individuals.

Here, we analyzed the factor structure and psychometric properties of the HRS in a large, multistudy sample containing multiple psychedelics and other psychoactive substances. In exploratory analyses, we also describe dose-response effects for different drugs and differences in HRS scores between classic psychedelics and dissociatives, empathogens, Δ^**9**^-tetrahydrocannabinol (THC), stimulants, and placebos. These analyses highlight the favorable psychometric and theoretical properties of the HRS in studies of psychedelics and other psychoactive substances.

## Methods and Materials

### Data Collection

**Figure 1:**
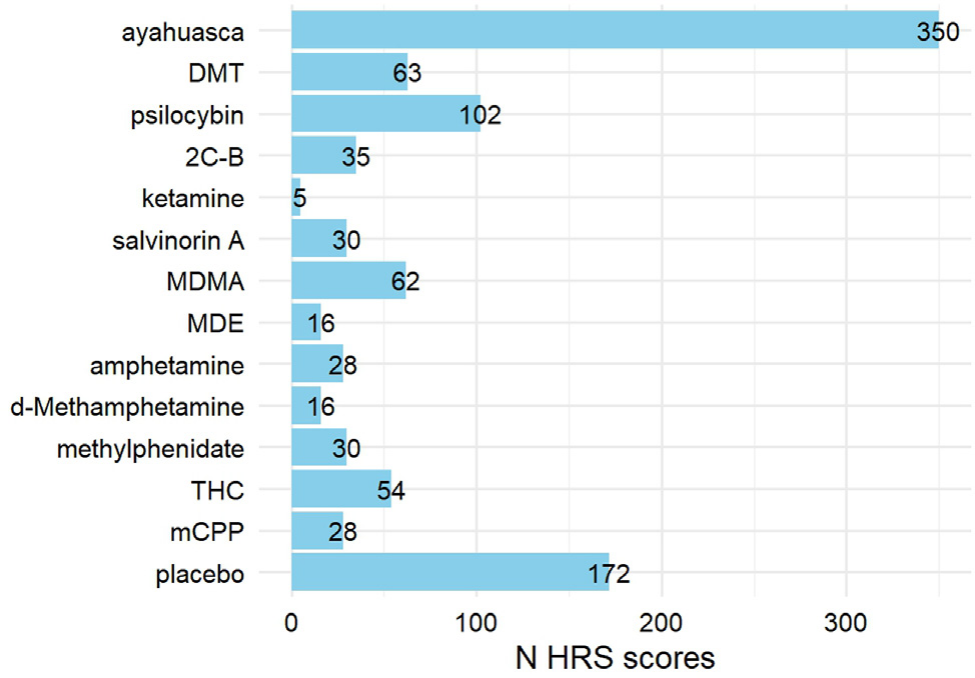
Number of Hallucinogen Rating Scale (HRS) questionnaires included in our dataset by drug, totaling 991 questionnaires from 599 unique individuals. mCPP, meta-chlorophenylpiperazine; THC, Δ^9^-tetrahydrocannabinol.

To gather data for our analyses, we contacted authors who had published studies using the HRS at any point through May 2023 (Figure S1). We searched for clinical studies in humans using the term “hallucinogen rating scale” in PubMed, which yielded 209 results. We identified 62 publications that had published unique data using the HRS. Authors were both able and willing to contribute raw data from 18 studies, which involved a total of 13 different psychoactive substances as well as placebo (Table 1 and Figure 1). All studies had been approved by an appropriate institutional review board, and all participants gave written informed consent before participating.

**Table 1:** Publications That Used the HRS Included in the Current Analysis

Reference	*N* HRS Scores (*N* Participants)	Substances and Doses	Placebo?	Population	% Female	Age, Years
Strassman and Qualls, 1994 (24)	39 (11)	DMT: 0.05–0.4 mg/kg (IV)^a^	No	Psychedelic users	9.10%	45 (4.92)
Strassman *et al.*, 1994 (25)	24 (12)	DMT: 0.04, 0.4 mg/kg (IV)^a^	No	Psychedelic users	8.30%	45 (4.92)
Bowdle *et al.*, 1998^b^ (14)	5 (5)	Ketamine: 0.5 mg/kg (IV)^a^	No	Healthy	0%	–
Grob, 1998^b^ (60)	52 (18)	MDMA: 0.25–2.5 mg/kg^a^	Yes	Healthy	33.33%^c^	20–62
Gouzoulis-Mayfrank *et al.*, 1999 (17)	63 (32)	d-Methamphetamine: 0.2, 0.4 mg/kg	Yes	Healthy	34.36%	34 (27–47)
		MDE: 2 mg/kg^a^				
		Psilocybin: 0.2 mg/kg^a^				
Riba *et al.*, 2001 (22)	71 (71)	Ayahuasca: various^a^	No	Ayahuasca users	46.48%^c^	36.59 (7.89)^c^
Riba *et al.*, 2001 (22)	56 (56)	Ayahuasca: various^a^	No	Ayahuasca users	14.29%^c^	26 (6.62)^c^
Tancer and Johanson, 2003 (18)	98 (14)	d-Amphetamine: 10, 20 mg	Yes	Healthy	50%	22.3 (18–31)
		mCPP: 0.5, 0.75 mg/kg^a^				
		MDMA: 1, 2 mg/kg^a^				
Griffiths *et al.*, 2006 (7)	60 (30)	Psilocybin: 0.43 mg/kg^a^	No	Healthy	61.11%	46 (24–64)
		Methylphenidate: 0.57 mg/kg				
Moreno *et al.*, 2006 (61)	29 (9)	Psilocybin: 0.025–0.3 mg/kg^a^	No	OCD	22.22%	40.9 (13.2)
Ballard *et al.*, 2012 (31)	81 (27)	THC: 7.5, 15 mg^a^	Yes	Healthy	44%	24.36 (4.56)
Caudevilla-Gálligo *et al.*, 2012 (12)	35 (35)	2C-B: 20 mg^a^	No	Healthy	23%	32.6 (6.53)
Addy *et al.*, 2015 (62)	60 (30)	Salvinorin A: 1017 μg^a^	Yes	Healthy	47%	39
Bouso *et al.*, 2016 (26)	158 (158)	Ayahuasca: various (mean 113 mL)^a^	No	Ayahuasca users	36.71%^c^	38.97 (9.0)^c^
Ross *et al.*, 2016 (8)	55 (29)	Psilocybin: 0.3 mg/kg^a^	Yes	Illness-related distress	62.10%	56.28 (12.93)
Palhano-Fontes *et al.*, 2019 (9)	29 (18)	Ayahuasca: 1 mL/kg^a^	Yes	Treatment-resistant depression	72%	42.03 (11.66)
		(0.36 mg/kg DMT)^a^				
Pasquini *et al.*, 2020 (63)	50 (25)	Ayahuasca: 1 mL/kg^a^	Yes	Healthy	53.49%	30.8 (8.4)
		(0.36 mg/kg DMT)^a^				
Apud *et al.*, 2022 (64)	26 (26)	Ayahuasca: various^a^	No	Ayahuasca users	38.46%^c^	36.96 (7.9)^c^
Total	991 (599)					

Doses were administered orally unless otherwise specified. Ages are shown as mean (SD) or range. Some discrepancies with published subject numbers are present due to missing data.

HRS, Hallucinogen Rating Scale; OCD, obsessive-compulsive disorder.

a Classical psychedelics and other drugs with psychedelic effects were used to analyze the factor structure of the HRS.

b HRS data from this source were obtained but had not been published previously.

c Individual participant data for age and sex were available for 5 studies.

### Statistical Analysis

We assessed the factor structure of the HRS using 745 HRS questionnaires for drugs with psychedelic properties. These included DMT, ayahuasca, psilocybin, and 2C-B, as well as drugs with some psychedelic-like or hallucinogenic properties, namely ketamine (28), MDMA (29), MDE (30), mCPP (29), and THC (31). We included 103 items, removing items 1 (time to effect) and 100 (dose you think you received) because they reflect study design and administration route rather than inherent drug properties. All statistical analyses were performed in R, version 4.3.2.

We randomly divided the sample into 2 halves balanced by drug, dose, and study site and then used one half for the EFA and the other for validation of the factor model in confirmatory factor analysis (CFA). EFA was performed using the psych package, version 2.3.6 (32), and CFA was performed using the lavaan package, version 0.6.16 (33). For the EFA, we configured a polychoric correlation matrix that we used to conduct an EFA with oblique (Promax) rotation based on robust estimations of diagonally weighted least squares. This method is appropriate for Likert-scale (i.e., ordinal) data, and robust estimation methods are appropriate when the assumption of normality is violated (34). Oblique rotation was chosen over orthogonal because factors obtained from psychological questionnaires are highly likely to correlate with each other. Careful consideration was given to choosing a factor retention method. We began with parallel analysis (35); however, this yielded some factors with only 2 items each. An alternative was chosen based on work using simulation studies, which suggests that for Likert scale data, retaining factors based on eigenvalues > 1 gives accurate results when factors do not correlate strongly, and the sample size is large (i.e., >100) (36). Because our data had these characteristics, we chose to retain factors with eigenvalues > 1, which yielded fewer factors that all contained at least 4 items. To maximize scale usefulness and model fit after EFA, we removed items for which all loadings were <0.30 and repeated the EFA until no such items remained (37).

Next, we compared the new factor model to the 2 previous models of HRS factor structure. Using the second half of the split dataset, we performed CFA based on robust estimations of diagonally weighted least squares to compare measures of fit between the 3 factor models. Criteria for good model fit were set as root-mean-square error of approximation <0.06, standardized root mean residual <0.08, comparative fit index and Tucker-Lewis Index >0.90, and χ^2^/*df* < 5 (38,39). Additionally, internal consistency of the factors was evaluated using Cronbach’s α and McDonald’s Ω. Criteria for acceptable consistency were set at >0.80 (40).

Using the entire dataset, we next explored changes in weighted factor scores for different drugs and doses compared with placebo using mixed-effects analyses of variance with each combination of drug and dose as a categorical predictor. Random effects were included for each study site and individual participant. Post hoc pairwise Wilcoxon tests were used to contrast each combination of drug and dose with placebo. The Benjamini-Hochberg procedure was applied to each level of testing to reduce the false discovery rate. Mixed-effects analyses of variance were calculated using R packages lme4, version 1.1.35.1 (41).

Finally, we divided substances into the categories of classic psychedelics, dissociatives, empathogens, THC, stimulants, or placebo. Classic psychedelics included DMT, ayahuasca, psilocybin, and 2C-B (42); dissociatives included salvinorin A and ketamine (43); empathogens included MDMA and MDE (28,30); and stimulants included amphetamine, methamphetamine, and methylphenidate. We used the same statistical strategy described above to investigate differences between classic psychedelics and other drug classes on each of the HRS factors.

## Results

### Factor Structure of the HRS

Bartlett’s test indicated the presence of correlations between items (*p* < .001), and the Kaiser-Meyer-Olkin test indicated good sampling adequacy of 0.90. Table S1 summarizes item means, skew, kurtosis, and difficulty. The final EFA revealed 8 factors (Figure S2) composed of 88 items (Table 2). These factors explained 49% of the common variance in scores, with individual factors explaining between 3% and 13% (Table S2). The model showed excellent fit (robust root-mean-square residuals = 0.04; a value < 0.05 indicates good fit). We chose factor names based on the dominant characteristics of each factor, and we kept 2 names (somaesthesia and volition) from the original clinical clusters.

**Table 2:** Factor Loadings of 88 Hallucinogen Rating Scale (HRS) Items Onto 8 Factors

Item	Factor 1 Vision	Factor 2 Meaningfulness	Factor 3 Dysphoria	Factor 4 Euphoria	Factor 5 Somaesthesia	Factor 6 Auditory and Minor Senses	Factor 7 Liking	Factor 8 Volition
64. Visual images, visions, or hallucinations	0.99^a^	0.02	0.03	−0.35	−0.06	0.08	−0.09	−0.12
57. Visual effects	0.94^a^	−0.10	−0.01	−0.10	−0.11	0.16	0.00	−0.08
68. Movement within visions/hallucinations	0.93^a^	0.02	0.05	−0.23	−0.11	0.11	−0.07	−0.13
65. Kaleidoscopic nature of images/visions/hallucinations	0.84^a^	0.08	0.01	−0.21	−0.12	0.08	−0.01	−0.17
66. Difference in brightness of visions	0.75^a^	0.07	−0.05	−0.18	−0.09	0.26	−0.01	−0.04
60. Change in acuity of vision / visual distinctiveness of objects	0.72^a^	−0.10	0.01	−0.07	0.00	0.32^a^	0.06	0.13
61. Visual field overlaid by patterns	0.71^a^	−0.18	0.10	−0.09	−0.01	0.13	0.23	0.05
67. Dimensionality of images/visions/hallucinations	0.71^a^	0.10	0.03	−0.11	−0.09	0.10	0.04	−0.06
58. Room looked different	0.69^a^	−0.19	0.14	0.05	−0.01	0.25	0.14	0.12
62. Vibration, jiggling, or other motion of the visual field	0.63^a^	−0.05	0.00	−0.15	0.14	0.20	0.15	0.06
59. Change in brightness of colors / objects in the room	0.59^a^	−0.09	0.05	0.00	0.09	0.34^a^	0.14	0.12
98. Intensity	0.59^a^	0.21	−0.04	0.05	0.15	−0.12	−0.06	0.11
82. Difference in feeling of reality	0.56^a^	0.25	−0.01	0.10	−0.07	0.02	−0.07	0.06
21. Feel removed, detached, separated from body	0.55^a^	0.07	0.15	0.04	−0.15	−0.04	0.06	0.02
6. Feel as if moving/falling/flying through space	0.55^a^	−0.04	0.14	0.12	0.02	−0.08	0.11	−0.03
5. Changes in sense of bodyweight	0.52^a^	−0.12	0.04	0.08	0.09	0.02	0.10	0.03
2. A “rush”	0.50^a^	−0.15	−0.14	0.18	0.30^a^	−0.02	−0.06	0.00
99. High	0.48^a^	−0.04	−0.07	0.26	0.08	−0.15	−0.12	0.07
83. Dreamlike nature of the experience	0.46^a^	0.14	0.09	−0.01	0.01	−0.16	−0.03	0.03
4. Body feels different	0.43^a^	−0.02	−0.15	0.17	0.29	−0.10	0.00	0.13
86. Change in rate of time passing	0.41^a^	0.22	0.06	0.13	0.05	0.01	−0.06	0.05
71. Sense of speed	0.38^a^	−0.11	0.22	0.11	0.15	−0.07	0.07	−0.04
80. Change in rate of thinking	0.33^a^	0.15	0.02	0.18	0.12	0.09	−0.10	−0.04
85. Insights into personal or occupational concerns	−0.01	0.91^a^	−0.16	−0.14	0.07	0.12	−0.15	−0.02
77. New thoughts or insights	0.21	0.76^a^	−0.15	0.03	−0.09	0.12	−0.14	0.04
84. Thoughts of present or recent past	0.01	0.69^a^	0.08	−0.23	0.10	0.16	−0.11	−0.04
27b. Forgiving yourself or others	−0.22	0.65^a^	0.07	0.01	−0.03	0.09	0.25	0.03
78. Memories of childhood	−0.11	0.64^a^	0.03	−0.23	0.26	0.13	0.00	0.00
33. Feel presence of numinous force, higher power, God	0.13	0.64^a^	−0.05	0.01	−0.10	−0.04	0.14	−0.02
43. Feeling of oneness with the universe	0.13	0.55^a^	−0.12	0.19	−0.12	0.12	0.19	−0.04
39. Feel like crying	0.03	0.52^a^	0.26	−0.09	0.06	−0.12	−0.06	−0.02
36. Sad	−0.05	0.52^a^	0.42^a^	−0.08	0.04	0.02	−0.10	−0.10
45. Feel reborn	0.02	0.51^a^	0.08	0.15	−0.04	−0.15	0.07	0.01
31a. Understanding of others' feelings	−0.05	0.51^a^	−0.03	0.07	0.11	0.15	0.21	0.05
40. Change in feelings of closeness to people in the room	0.07	0.48^a^	0.00	0.11	0.04	0.20	0.00	0.16
36a. Loving	−0.24	0.47^a^	0.02	0.07	0.15	0.05	0.33^a^	0.10
79. Feel like a child	−0.07	0.45^a^	0.09	0.13	0.06	0.05	0.06	0.04
41. Change in “amount” of emotions	0.10	0.42^a^	0.07	0.36^a^	−0.02	0.17	−0.11	0.05
81. Change in quality of thinking	0.32^a^	0.42^a^	−0.11	0.21	−0.01	0.09	−0.13	0.02
27a. Self-accepting	−0.02	0.42^a^	−0.08	0.22	−0.15	0.06	0.31^a^	−0.01
76. Change in strength of sense of self	0.22	0.42^a^	0.03	0.28	−0.08	0.11	−0.07	−0.03
74. Contradictory feelings at the same time	0.02	0.35^a^	0.28	0.10	0.02	0.17	−0.15	0.05
53. Sense of silence or deep quiet	0.15	0.31^a^	0.24	0.06	0.00	−0.09	0.20	−0.09
27. Panic	0.04	−0.03	0.85^a^	−0.01	0.00	−0.19	0.05	0.04
38. Despair	−0.05	0.10	0.78^a^	0.06	0.03	−0.06	−0.03	−0.04
26. Frightened	0.05	0.18	0.66^a^	−0.10	0.11	−0.11	−0.12	0.02
75. Sense of chaos	0.24	−0.06	0.58^a^	0.08	0.04	−0.07	−0.10	0.04
25. Anxious	0.00	−0.01	0.52^a^	0.13	0.22	−0.18	−0.22	0.05
88. How sane did you feel?	0.09	0.06	0.45^a^	0.17	−0.09	0.13	−0.10	0.05
44. Feel isolated from people and things	0.12	−0.07	0.45^a^	0.08	0.00	0.05	−0.11	0.01
70. Dead or dying	0.11	0.25	0.43^a^	−0.10	0.09	−0.23	0.10	−0.01
87. Unconscious	0.16	−0.15	0.42^a^	0.11	−0.08	0.14	−0.01	−0.01
37. Euphoria	−0.22	0.09	0.08	0.88^a^	−0.12	0.06	0.07	−0.08
30. Excited	0.05	−0.10	0.13	0.74^a^	−0.01	−0.17	0.13	−0.04
29. Feel like laughing	−0.10	−0.01	0.02	0.70^a^	−0.03	0.16	0.11	−0.05
35. Happy	−0.13	0.13	−0.02	0.62^a^	−0.05	0.08	0.43^a^	−0.04
20. Sexual feelings	−0.27	−0.08	0.06	0.56^a^	0.07	0.25	0.05	0.07
31. Awe, amazement	0.20	0.32^a^	0.16	0.44^a^	−0.24	−0.12	0.02	−0.03
42. Emotions seem different than usual	0.13	0.26	−0.01	0.41^a^	0.00	0.18	−0.15	0.00
10. Shaky feelings inside	−0.03	0.23	−0.13	−0.21	0.92^a^	−0.06	−0.14	−0.03
11. Body shake/tremble on the outside	0.02	0.15	0.03	−0.17	0.78^a^	−0.05	−0.09	−0.04
12. Feel heart beating	−0.05	−0.02	0.04	0.00	0.54^a^	0.17	0.07	−0.06
3. Change in salivation	−0.11	−0.14	0.12	0.20	0.43^a^	0.04	0.08	0.04
9. Pressure or weight in chest or abdomen	−0.05	0.13	0.21	−0.11	0.40^a^	0.04	0.06	−0.04
13. Heart skipping beats	0.04	−0.11	0.31^a^	−0.04	0.40^a^	0.07	0.11	−0.06
90. Change in effort of breathing	−0.08	0.08	0.20	0.03	0.39^a^	0.02	0.08	−0.04
8. Electric/tingling feeling	0.10	−0.05	−0.09	0.26	0.38^a^	−0.02	−0.02	−0.02
16. Physically restless	−0.12	0.00	0.27	0.21	0.31^a^	0.08	−0.15	0.01
54. Sounds in room sound different	0.14	0.20	−0.15	0.11	0.04	0.71^a^	−0.11	−0.01
34. Change in feelings about sounds in the room	0.17	0.19	−0.10	0.12	−0.01	0.67^a^	−0.17	−0.04
55. Difference in distinctiveness of sounds	0.23	0.24	−0.08	0.05	−0.05	0.66^a^	−0.11	−0.01
52. A sound or sounds accompanying the experience	0.24	0.11	−0.07	−0.06	0.16	0.38^a^	−0.06	−0.02
22. Change in skin's sensitivity	0.16	−0.12	−0.11	0.17	0.35^a^	0.36^a^	0.01	0.08
63. Visual synesthesia	0.28	0.12	0.06	0.06	−0.09	0.32^a^	0.00	−0.09
50. An odor	0.08	0.21	0.09	−0.13	0.24	0.32^a^	0.05	−0.04
28. At ease	0.06	0.09	−0.03	0.07	−0.06	−0.14	0.65^a^	0.00
15. Physically comfortable	0.05	−0.21	0.15	0.26	−0.08	−0.06	0.61^a^	−0.02
48. How soon would you like to repeat the experience?	0.06	0.05	−0.19	−0.09	0.10	−0.18	0.55^a^	−0.01
49. Is this an experience you would like to have regularly?	0.07	0.22	−0.17	−0.08	0.03	0.04	0.52^a^	0.00
47. Like the experience	0.15	0.21	−0.30	0.22	0.14	−0.08	0.50^a^	−0.06
46. Satisfaction with the experience	0.18	0.27	−0.33	0.30	0.08	−0.05	0.35^a^	−0.11
95. Able to move around if asked to do so	0.40^a^	−0.03	0.02	−0.27	−0.11	−0.18	0.17	0.63^a^
91. Able to follow the sequence of events	−0.07	−0.05	0.04	0.01	−0.01	−0.04	0.00	0.62^a^
93. Able to focus attention	−0.04	−0.06	−0.02	0.09	0.04	0.01	−0.16	0.60^a^
94. In control	0.13	0.00	−0.02	0.16	−0.02	−0.09	−0.03	0.58^a^
32. Safe	−0.15	0.04	−0.01	0.00	−0.10	0.21	−0.08	0.53^a^
96. Awareness of external situation	−0.04	0.26	0.12	−0.24	−0.14	0.01	0.12	0.49^a^
92. Able to “let go”	−0.30	0.09	−0.05	−0.11	0.11	−0.01	−0.03	0.48^a^

Factors are arranged from left to right in order of variance explained. Items are arranged by loading strength on the factor to which they were assigned.

a Loadings > 0.3.

### Validation of Factor Model and Comparison to Previous Models

Using the other half of our dataset, we used CFA to test the validity of the new 8-factor model and compare it with the original clinical clusters (25), as well as to a subsequent factor model from an ayahuasca study (26). All models showed good to excellent internal consistency, with the new model having the highest consistency (Table S2). For the 8-factor model, the comparative fit index, Tucker-Lewis Index, and standardized root mean residual indicated good model fit (Table 3). For the original clinical clusters, the comparative fit index and Tucker-Lewis Index values indicated good model fit, while the other measures were outside cutoffs for acceptable fit. The model fit for the solution proposed by Bouso *et al.* (26) was initially too poor to calculate measures of fit. However, when performing CFA using only data from that study, we found an identifiable solution with acceptable fit (root-mean-square error of approximation = 0.078), similar to what was reported previously. Thus, this previous factor structure was still identifiable in its original ayahuasca dataset, but not in the overall dataset. Overall, the new 8-factor model could be validated in the second half of the dataset and showed superior measures of fit compared with previous models.

**Table 3:** Comparison of Model Fit for 2 Factor Models After Confirmatory Factor Analysis Based on Robust Measures of Weighted Least Squares

Factor Model	*df*	χ^2^	χ^2^/*df*	CFI	TLI	RMSEA	SRMR
Clinical Clusters [Strassman *et al.* (25)]	2195	4298.14	1.96	0.921	0.918	0.077	0.088
Eight Factors (Current Solution)	3712	5849.62	1.58	0.937	0.935	0.066	0.078

*n* = 745.

CFI, comparative fit index; RMSEA, root-mean-square error of approximation; SRMR, standardized root mean residual; TLI, Tucker-Lewis fit index.

Next, we calculated Spearman correlations between factors to examine the relationship between the 8 new factors and the original clinical clusters (Figure S3). The factors of somaesthesia and volition correlated highly and specifically with the clusters of the same name. Most factors showed strong correlations with multiple clinical clusters.

### Factor Scores by Drug and Dose

Next, we investigated how well the 8 new HRS factors differentiated various drugs from placebo. All 8 factors showed significant differences between drugs (Table S3). Post hoc pairwise Wilcoxon tests were used to compare each drug and dose combination with placebo on each of the 8 factors (Table S4).

**Figure 2:**
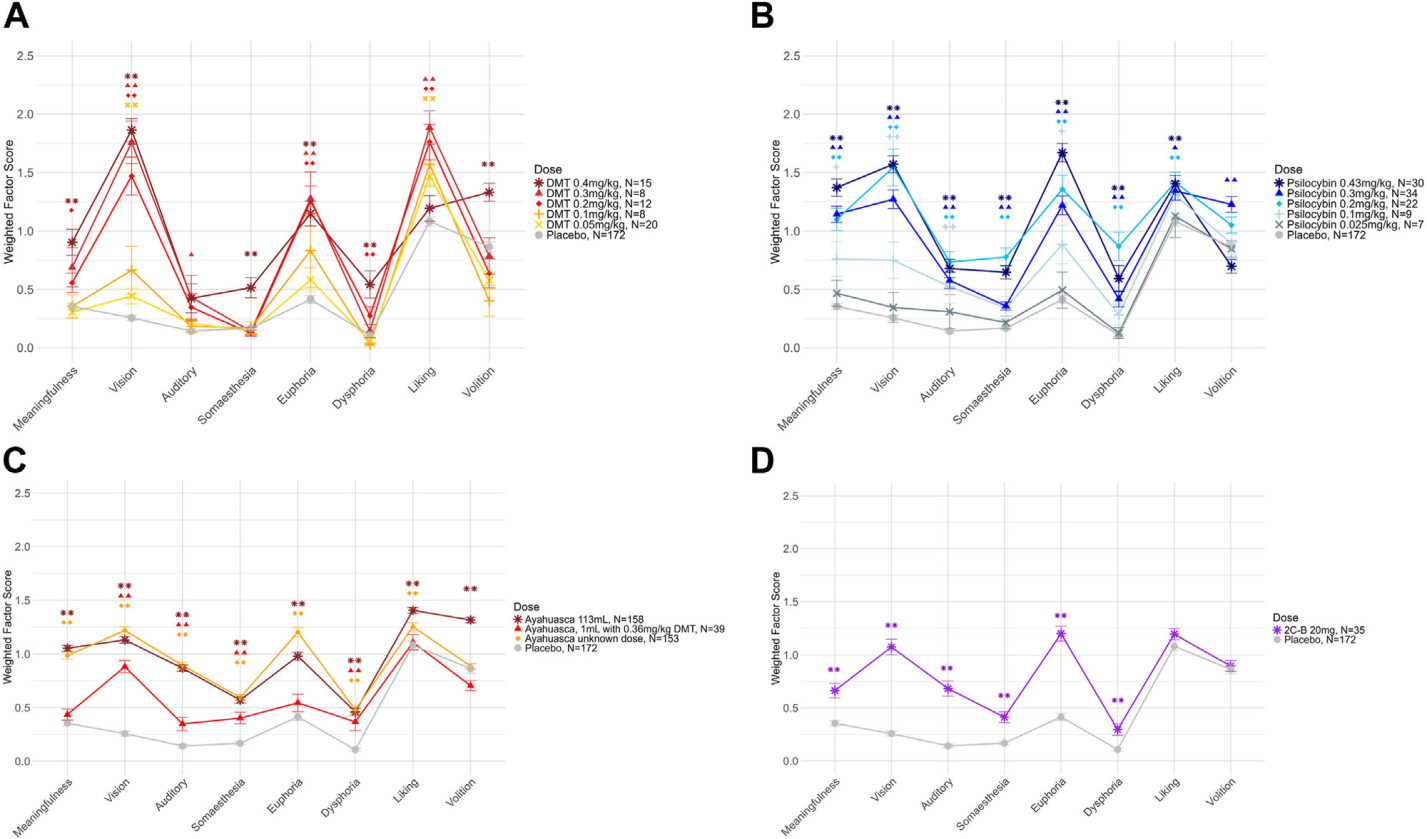
Average weighted Hallucinogen Rating Scale factor scores for different doses of the classic psychedelics **(A)** DMT, **(B)** psilocybin, **(C)** ayahuasca, and **(D)** 2C-B. Error bars show the SEM. Pairwise Wilcoxon tests with Benjamini-Hochberg correction were used to test differences between placebo and each dose. ∗∗*p* < .01, ∗*p* < .05; symbols represent contrasts between each dose and placebo.

Every classic psychedelic had significant effects on the new HRS factors at fully psychoactive doses. Most doses of DMT (Figure 2A) successively increased scores on vision, euphoria, and liking, although the highest dose of 0.4 mg/kg notably no longer showed a significant effect on liking. Only the highest dose significantly increased volition and somaesthesia, and only the 2 highest increased meaningfulness and dysphoria scores. Psilocybin doses of at least 0.2 mg/kg significantly increased scores on all 8 factors except for volition, which only increased at 0.3 mg/kg (Figure 2B). Smaller doses of psilocybin also had subtle but significant effects on the factors vision, auditory and minor senses, meaningfulness, and euphoria. The single dose of 2C-B (20 mg) increased HRS scores on all factors except liking and volition (Figure 2D).

**Figure 3:**
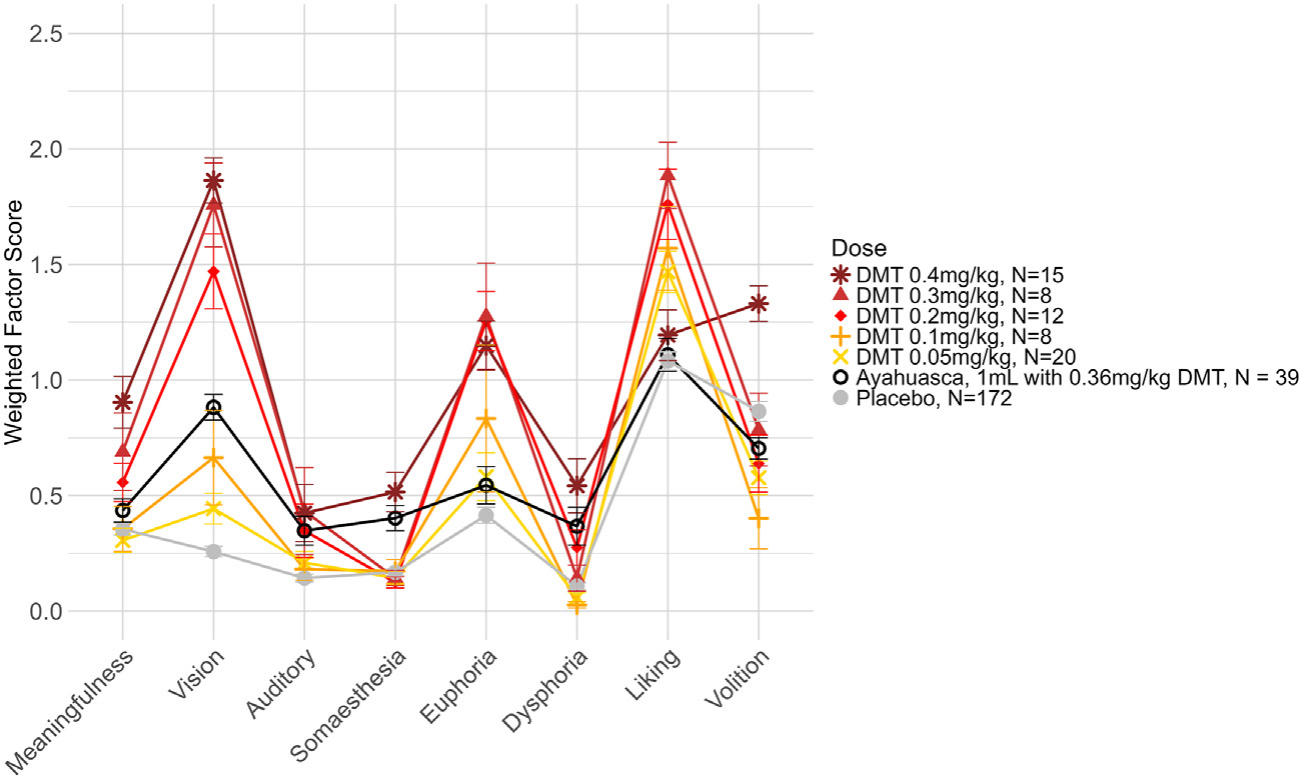
Average weighted Hallucinogen Rating Scale factor scores for different doses of the classic psychedelic DMT, with ayahuasca shown for comparison. Error bars show the SEM.

For ayahuasca, the exact amounts of DMT and β-carbolines were not always known. One study reported an average dose of 113 mL, which was sufficient to increase HRS scores on all factors (Figure 2C). This dose had effects that were visually similar to the average of the unknown doses from other studies. Additionally, 2 studies used a dose of 1 mL/kg ayahuasca with a DMT content of 0.36 mg/kg. This dose was sufficient to significantly increase scores on the factors vision, auditory and minor senses, somaesthesia, and dysphoria. Additionally, the profile of effects for 0.36 mg/kg DMT in 1 mL/kg ayahuasca in a ceremonial setting did not fully resemble the profile of any intravenous DMT dose in a clinical setting. While the perceptual effects were similar to a dose of 0.1 to 0.2 mg/kg intravenous DMT, ayahuasca’s effects on other factors more closely resembled higher (somaesthesia, dysphoria) or lower (euphoria, liking) doses of intravenous DMT (Figure 3).

**Figure 4:**
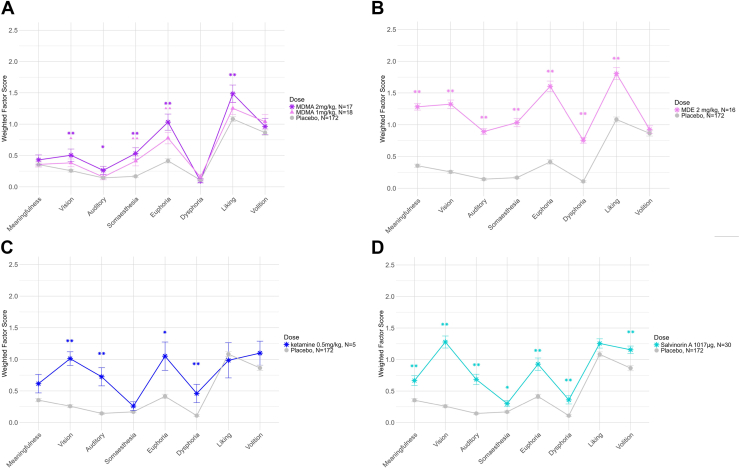
Average weighted Hallucinogen Rating Scale factor scores for empathogens and dissociatives, namely **(A)** MDMA, **(B)** MDE, **(C)** ketamine, and **(D)** salvinorin A. Error bars show the SEM. Pairwise Wilcoxon tests with Benjamini-Hochberg correction were used to test differences between placebo and each dose. ∗∗*p* < .01, ∗*p* < .05; symbols represent contrasts between each dose and placebo.

MDMA doses of 1 mg/kg and 2 mg/kg significantly increased scores on the factors euphoria, somaesthesia, and vision (Figure 4A). The higher dose also significantly increased liking and auditory and minor senses scores. Other doses of MDMA increased scores on some factors (Table S4), but the small samples per dose prohibited clear visualization of dose-response effects. Regarding MDE, all factors except volition showed a significant increase at a dose of 2 mg/kg (Figure 4B). Salvinorin A significantly increased scores on all factors except liking (Figure 4D), while ketamine increased scores on vision, auditory and minor senses, euphoria, and dysphoria (Figure 4C).

**Figure 5:**
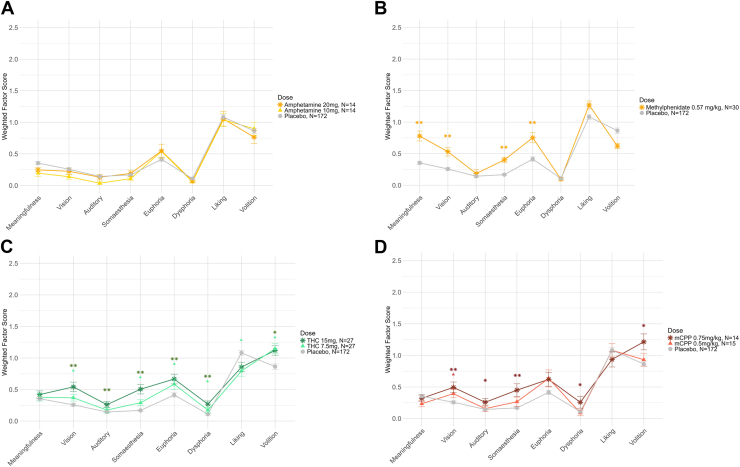
Average weighted Hallucinogen Rating Scale factor scores for the stimulants **(A)** amphetamine and **(B)** methylphenidate, as well as **(C)** Δ^9^-tetrahydrocannabinol (THC) and **(D)** meta-chlorophenylpiperazine (mCPP). Error bars show the SEM. Pairwise Wilcoxon tests with Benjamini-Hochberg correction were used to test differences between placebo and each dose. ∗∗*p* < .01, ∗*p* < .05; symbols represent contrasts between each dose and placebo.

Among stimulants, amphetamine did not increase any factor scores (Figure 5A), and methylphenidate increased scores on meaningfulness, vision, somaesthesia, and euphoria (Figure 5B). Methamphetamine scores were unexpectedly high but not easily interpretable due to the high placebo scores in the study from which they originated (Table S4) (17). Finally, THC and mCPP both showed dose-dependent effects on some HRS factors (Figure 5C, D). At least 1 dose of THC showed significant increases on all factors except for meaningfulness, while the highest dose of mCPP increased vision, auditory and minor senses, somaesthesia, dysphoria, and volition.

### Comparison of Classic Psychedelics to Other Substance Classes

**Figure 6:**
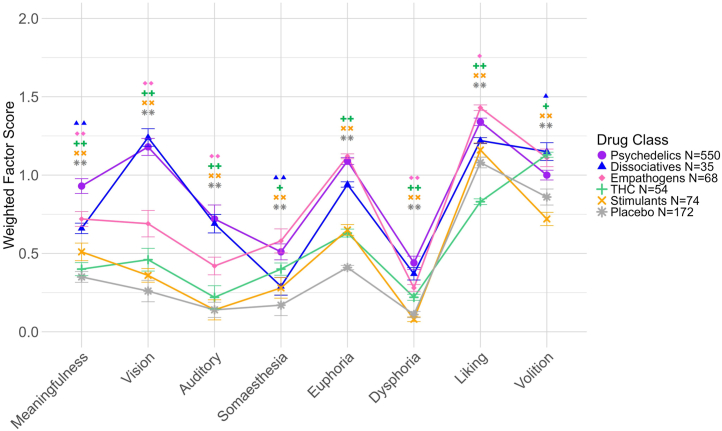
Weighted average Hallucinogen Rating Scale factor scores for psychoactive doses of 5 substance classes and placebo. Error bars show the SEM. Pairwise Wilcoxon tests with Benjamini-Hochberg correction were used to test differences between psychedelics and other substance classes on each of the 8 factors. ∗∗*p* < .01, ∗*p* < .05; symbols represent contrasts between psychedelics and each other substance class.

We pooled the data by drug class (classic psychedelics, dissociatives, empathogens, THC, stimulants, and placebo) to investigate the discriminant validity of the HRS for different drugs. All 8 factors showed differences between drug classes (Table S5). Post hoc pairwise Wilcoxon tests indicated that scores were significantly higher for psychedelics than for placebo, stimulants, and THC on all factors (Figure 6; summary statistics shown in Table S6). Scores were also significantly higher for psychedelics than for empathogens on 5 of 8 factors and than for dissociatives on 3 of 8 factors.

The meaningfulness factor significantly distinguished psychedelics from all other substance classes. A closer examination of meaningfulness using pairwise Wilcoxon tests showed that all items could differentiate psychedelics from placebo and at least one other drug class, and 4 items significantly differentiated classic psychedelics from all other drugs, namely “contradictory feelings at the same time,” “memories of childhood,” “thoughts of present or recent past,” and “insights into personal or occupational concerns” (Table S7).

## Discussion

We analyzed the factor structure and psychometric properties of the HRS in a large, multistudy sample including 13 different psychedelics and other psychoactive substances. We presented an 8-factor solution comprising 88 of the original 105 HRS items. which shows good to excellent model fit and internal consistency. The 8 factors are 1) vision, 2) meaningfulness, 3) dysphoria, 4) euphoria, 5) somaesthesia, 6) auditory and minor senses, 7) liking, and 8) volition. In addition, we included enough participants to meet conservative recommendations for sample size in EFA. Model fit and internal consistency were equal or superior to previous factor models of the HRS, and the 8 factors are conceptually coherent in addition to their favorable statistical properties. The full HRS, including scoring instructions, can be found in English and German in the Supplement.

The vision factor contained all items referring to visual changes, including simple and complex visual imagery and changes in the perception of movement, light, and color. The highest vision scores were for psychedelics and dissociatives, increasing by dose and consistent with their perceptual effects (1). Interestingly, items describing changes in nonvisual sensory perception loaded together on the factor auditory and minor senses. Vision and hearing have also been treated as separate factors in other psychedelic rating scales (44). This separation may reflect the prominence of vision both in human perception overall and in psychedelic experiences.

The meaningfulness factor contained items related to personal autobiography, emotionally charged psychological insight, and mystical-type experiences. Psychedelics can enhance autobiographical memory and alter one’s perspective on past events (45,46), and the related phenomenon of psychological insight is also characteristic of their effects (47,48). Three items related to autobiographical memory and insight distinguished psychedelics from all other drugs (memories of childhood, thoughts of present or recent past, insights into personal or occupational concerns). Additionally, psychedelic-induced insight often involves “emotional breakthroughs” (49), reflected in emotionally charged items (e.g., feel like crying, loving, forgiving yourself or others) on the meaningfulness factor. Some aspects of mystical-type experiences were also found in this factor, including a sense of unity or sacredness (e.g., items 43, 53, 76), paradoxicality (item 74), and greater connectedness to others or to oneself (e.g., items 27a, 31a, 40) (50,51). The item assessing “contradictory feelings at the same time,” i.e., paradoxicality, was also one of the 4 items that discriminated psychedelics from all other drug classes, supporting recent work identifying paradoxicality as a key psychedelic effect (51). Finally, psychedelics characteristically enhance feelings of meaning, giving this factor its name (52). Personal insight and mystical-type experiences are felt to be highly meaningful, a characteristic of psychedelic drugs that is associated with lasting positive changes in well-being (7,49). Unique among the 8 factors, higher scores on meaningfulness set psychedelics apart from all other drug classes.

The HRS differentiated between 2 related but distinct types of positive affect. Euphoria contained items related to highly pleasurable emotional states, including laughter, sexual feelings, and feeling happy, excited, or in awe. Many people who take psychedelics, including healthy study participants, are motivated by the intense euphoric effects represented in the euphoria factor (53,54). Items in the liking factor, although also positively valanced, were related to participants’ feelings of comfort and satisfaction with the experience, as well their desire to repeat it. Liking was also the only factor on which empathogens scored significantly higher than psychedelics. Additionally, liking showed relatively high scores in the placebo conditions, probably because it contains items that are likely to be rated highly even when no substances are given (e.g., items 28, 46). Thus, it may be less sensitive to subtle changes than the other factors.

The factor dysphoria encompasses negative mood states, particularly fearful ones, which like visual effects and mystical-type experiences nearly always appear as a factor on psychedelic rating scales (44,51). Dysphoric psychedelic states, sometimes called “bad trips,” have also been characterized in detail by the Challenging Experience Questionnaire (55). The Challenging Experience Questionnaire comprises 7 factors, several of which (sensations of fear, isolation, insanity, and death) are clearly represented in the dysphoria factor of the HRS. Additionally, the somaesthesia factor reflects physical symptoms that are often, but not always, experienced as unpleasant, including involuntary muscle shaking, restlessness, and changes in heart rate, salivation, and breathing. Somaesthesia correlated more highly with dysphoria (ρ = 0.63) than with any other factor, and it may reflect symptoms of sympathetic activation.

Finally, the volition factor contained items describing control over one’s body and mind, as well as situational awareness and feelings of safety. The degree of volitional control over one’s body, emotions, perceptions, and thoughts is an important variable in psychedelic experiences, which are often associated with profound losses of control at high doses (56). In research settings, people are often coached into letting go of control in order to reduce the chance of anxious reactions (57). Volition behaved somewhat differently than the other factors and was often the last to have significantly different scores from placebo with ascending dose. It was also the only factor on which classic psychedelics scored significantly lower than THC and dissociatives.

The analysis of differences between drugs and doses is only exploratory given the variation in sample sizes and design between studies. Overall, however, the patterns of effects are consistent with what is already known about the drugs analyzed. Classic psychedelics, including DMT, ayahuasca, psilocybin, and 2C-B, all significantly increased scores on the 8 HRS factors, and clear dose-response patterns were observed when a range of doses was available. Scores on all 8 factors were significantly different for classic psychedelics than for the placebos, stimulants, and THC. Additionally, psychedelics differed from empathogens on all factors except somaesthesia, euphoria, and volition and from dissociatives on the factors of meaningfulness, somaesthesia, and volition. Classic psychedelics also showed higher scores on the meaningfulness factor than all other drug classes.

Taken together, these results demonstrate that the HRS is a reliable and valid rating scale for assessing a broad range of subjective psychedelic drug effects. It parses into 8 factors that capture unique psychedelic phenomena and characterize the effects of other substances with overlapping properties. Additionally, the factors and items included on the HRS make it one of the broadest measures of psychedelic drug effects. It quantifies several important properties that are not well represented in other common rating scales, including physical or bodily sensations, autobiographical memory, self-control, and perception of sounds, touch, smell, and taste. At the same time, major categories of psychedelic drug effects identified with other rating scales also appear on the HRS factors, including visual changes, positive and negative effects on mood, and aspects of mystical-type experiences. Notably, the HRS neither emphasizes nor ignores mystical-type experiences, probably due to its basis in phenomenological interviews rather than any specific theory of psychedelic drug effects. This is one difference from other general psychedelic rating scales, including various iterations of the Altered States of Consciousness Rating Scale and the Mystical Experience Questionnaire, which were strongly influenced by Stace’s concept of mystical experience (44,51). Aside from the clear application to studies of psychedelic drugs, the HRS may also be useful in studies of novel psychoactive substances with potential psychedelic properties, as well as studies of non–drug-altered states of consciousness such as meditative states, breathwork, or near-death experiences.

### Limitations

Our data were limited by the specific drugs that we were able to include. Both the range of doses and sample size for specific doses were sometimes quite small. Therefore, our findings on differences between different drugs and doses are preliminary, and future studies with larger samples and a larger range of doses are needed to make proper comparisons. The current dataset also contains variability in the context in which the substances were given. Our dataset included multiple languages, and translations can affect participants’ responses. Most studies involved research or clinical settings, but some ayahuasca studies took place in ceremonial environments. Drug effects vary strongly based on contextual factors, and this is particularly true for psychedelics (58). The advantage of including multiple study sites is that some of the site-specific variance in the setting may have been averaged out, making drug effects easier to observe. However, our results should not be taken as incontrovertible evidence for differences between these substances in all settings. They mostly reflect differences that arise within a research setting, and the pattern of effects may change in other settings. Finally, the HRS could be updated in the future by removing items that are not sensitive to drug effects or do not load onto factors and by adding items assessing relevant experiences not yet included. For example, reports of encounters with so-called entities are not included in any psychedelic rating scale, despite their reported prevalence in DMT experiences (59). The results of the current analyses also still require confirmation using CFA in an independent dataset.

### Conclusions and Future Directions

This analysis demonstrates that the HRS, in its current form, resolves into 8 factors that are conceptually sound and demonstrate a good degree of fit as well as good to excellent internal consistency. Given its strong psychometric properties and intuitive factors, the HRS remains a valuable tool for measuring psychedelic drug effects in a relatively theory-free manner, using basic question formulations that parse psychedelic experiences into their fundamental components. Future studies should assess the convergent and discriminant validity of the 8 HRS factors as well as their relationship to long-term outcomes resulting from psychedelic experiences or psychedelic-assisted therapy.

## Supplementary Material

Supplemental Methods, Figures S1–S3, and Tables S1–S7

Hallucinogen Rating Scale Version 4 English

Hallucinogen Rating Scale Version 4 German

Key Resources Table
