# Supplementary material for: The Hallucinogen Rating Scale: Updated Factor Structure in a Large, Multistudy Sample
Source: Biol Psychiatry Glob Open Sci. 2024 Dec 19;5(2):100436. doi: 10.1016/j.bpsgos.2024.100436 (PMC11804565; doi:10.1016/j.bpsgos.2024.100436)
Supplement: Supplemental Methods, Figures S1–S3, and Tables S1–S7 [file mmc1.pdf]

SUPPLEMENTARY INFORMATION

The Hallucinogen Rating Scale: Updated Factor Structure in a Large, Multi-Study Sample

Calder *et al.*

Supplementary Methods: HRS Translation into German

The translation process of the HRS from English into German followed the scientific guidelines for questionnaire translation and cross-cultural adaptation (Beaton et al., 2000; Fenn et al., 2020). Following the guidelines, the HRS was forward and back-translated from English into German by native speakers who were also fluent in the target language. All discrepancies were discussed, and the outcomes of these discussions documented in detail at every stage of the translation process.

First, two native German speakers produced independent German translations of the HRS. Subsequently, the two versions were compared and discrepancies between them were discussed and resolved. To guarantee the accuracy of the newly translated German version, two expert English speakers who were not previously familiar with the questionnaire independently back-translated the HRS into English. Again, discrepancies between the two versions were discussed and resolved. Items in the new back-translation that did not align with the original English version were discussed by an expert panel comprising the four translators and an additional researcher with expertise in the subject matter. The German version was modified accordingly.

Supplementary References:

Beaton, D. E., Bombardier, C., Guillemin, F., & Ferraz, M. B. (2000). Guidelines for the process of cross-cultural adaptation of self-report measures. *Spine*, *25*(24), 3186–3191. https://doi.org/10.1097/00007632-200012150-00014

Fenn, J., Tan, C.-S., & George, S. (2020). Development, validation and translation of psychological tests. *BJPsych Advances*, *26*(5), 306–315. https://doi.org/10.1192/bja.2020.33

Supplementary Figures

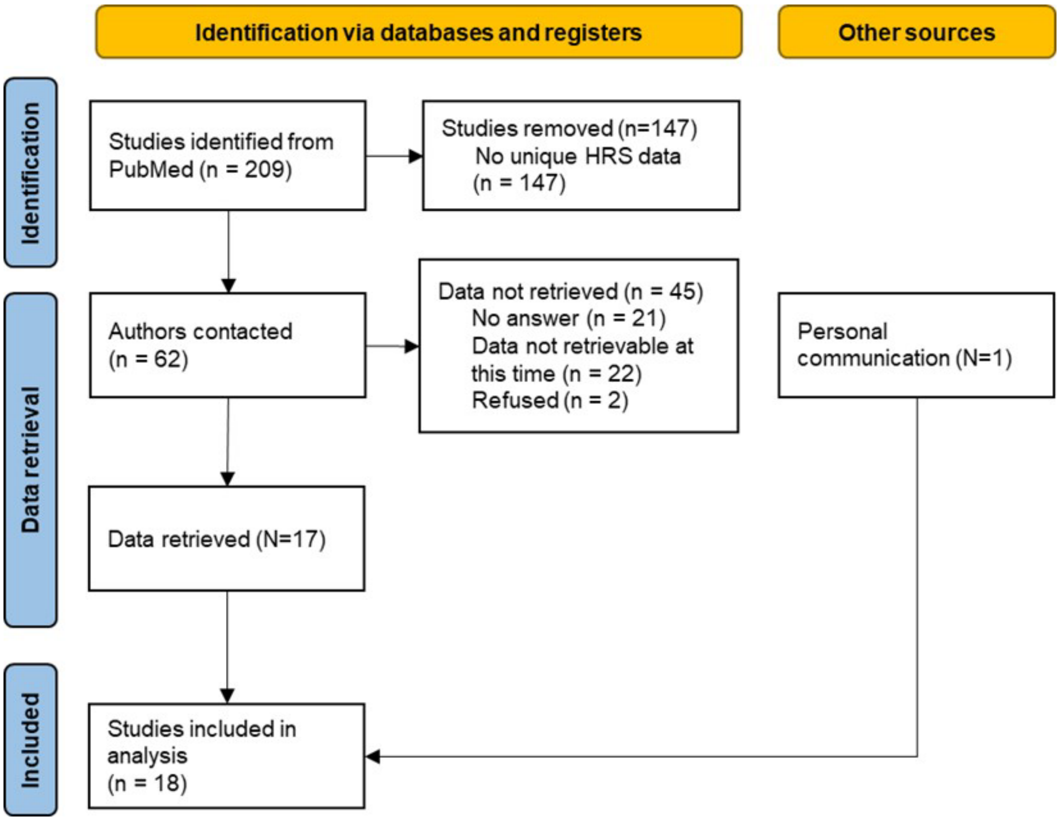

Figure S1. PRISMA diagram showing the process of data retrieval from studies including HRS data after drug administration.

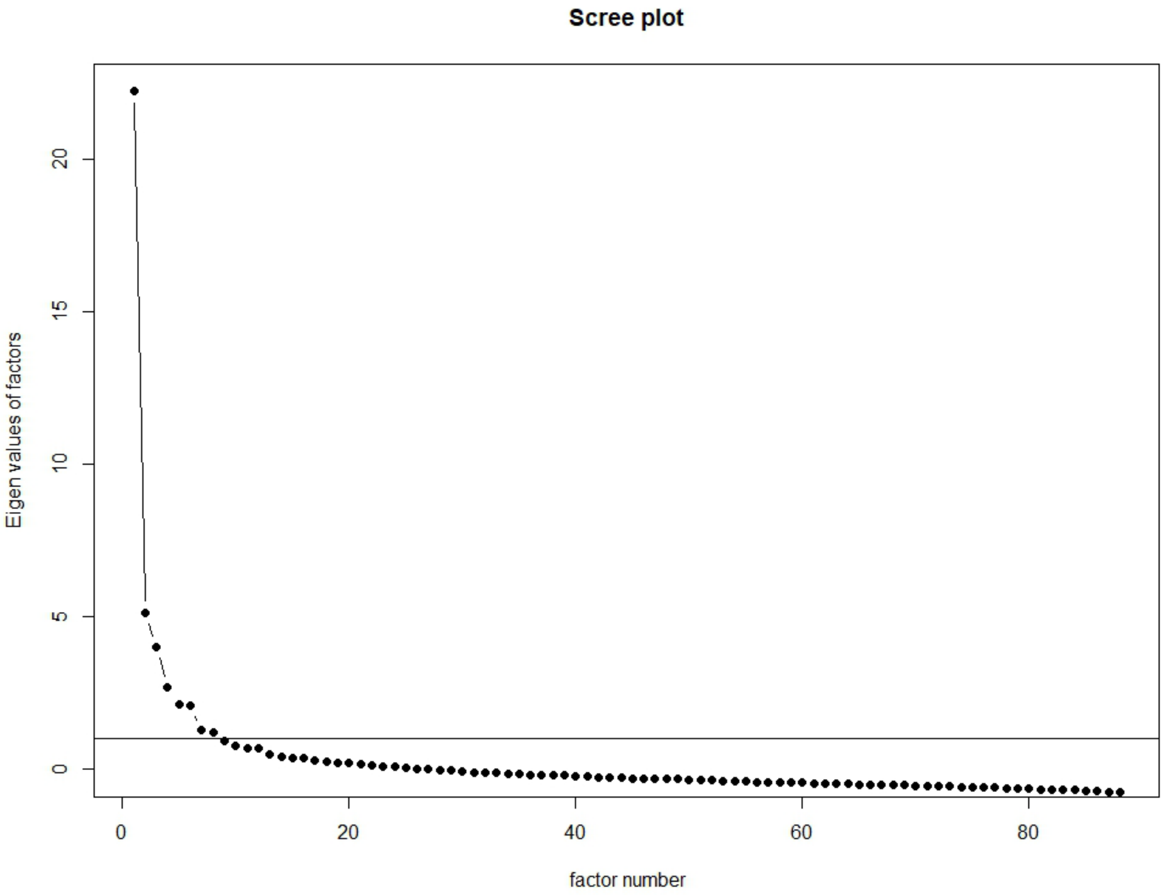

Figure S2. Scree plot displaying eigenvalues for the 88 HRS items used in the final exploratory factor analysis. Eight factors showed an eigenvalue > 1.

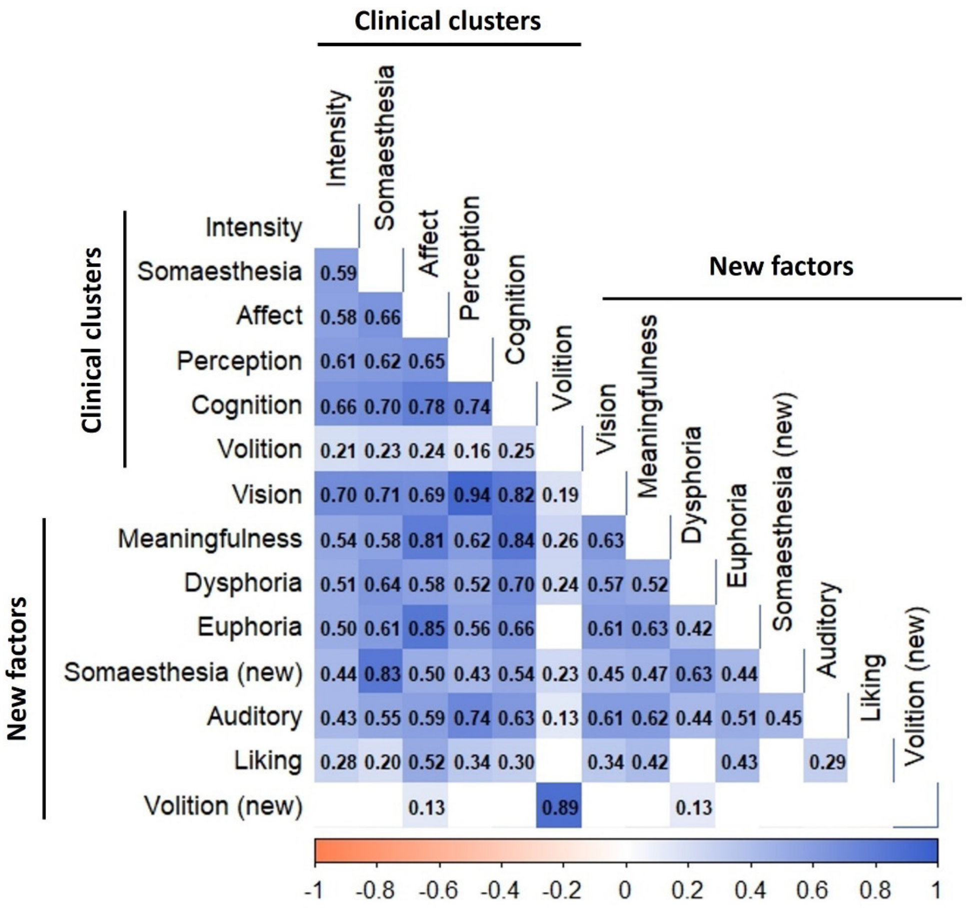

Figure S3. Spearman correlation coefficients for the new eight factor model and the six original clinical clusters. Only significant correlations (Bonferroni corrected p <0.05) are shown.

Supplementary Tables

Table S1: Characteristics of 103 HRS items included in the analysis.

Item	Mean	SD	Skew	Kurtosis	Difficulty (mean / range)
2. A "rush"	1.97	1.22	-0.02	-0.82	0.39
3. Change in salivation	1.18	1.19	0.75	-0.35	0.24
4. Body feels different	2.35	1.17	-0.37	-0.56	0.47
5. Changes in sense of bodyweight	1.44	1.25	0.42	-0.88	0.29
6. Feel as if moving/falling/flying through space	1.17	1.3	0.81	-0.54	0.23
7. Change in body temperature	1.48	1.19	0.21	-1.07	0.37
8. Electric/tingling feeling	1.33	1.19	0.53	-0.71	0.27
9. Pressure or weight in chest or abdomen	0.76	1.05	1.28	0.78	0.19
9a. Physically loose, limber or flexible	1.35	1.25	0.43	-1.06	0.34
10. Shaky feelings inside	1.02	1.14	0.93	-0.03	0.2
11. Body shake/tremble on the outside	1.01	1.2	0.95	-0.17	0.2
12. Feel heart beating	0.96	1.09	0.88	-0.35	0.24
13. Heart skipping beats	0.35	0.72	2.37	5.71	0.09
14. Nausea	0.82	1.14	1.32	0.76	0.21
15. Physically comfortable	2.5	1.15	-0.49	-0.31	0.5
16. Physically restless	1.3	1.23	0.63	-0.58	0.26
17. Flushed	0.64	0.89	1.3	1	0.16
18. Urge to urinate	0.88	1.07	1.07	0.27	0.22
19. Urge to move bowels	0.44	0.82	2.26	5.24	0.11
20. Sexual feelings	0.72	1	1.3	0.82	0.18
21. Feel removed, detached, separated from body	1.36	1.36	0.66	-0.75	0.27
22. Change in skin's sensitivity	1.42	1.25	0.38	-0.98	0.28
23. Sweating	0.66	0.95	1.38	1.04	0.17
24. Headache	0.41	0.76	2.12	4.68	0.1
25. Anxious	0.98	1.12	1.05	0.31	0.2
26. Frightened	0.7	1.04	1.54	1.71	0.14
27. Panic	0.41	0.89	2.58	6.57	0.08
27a. Self-accepting	2.23	1.32	-0.46	-0.92	0.45
27b. Forgiving yourself or others	1.87	1.51	-0.02	-1.45	0.37
28. At ease	2.46	1.21	-0.45	-0.57	0.49
29. Feel like laughing	1.78	1.33	0.17	-1.02	0.36
30. Excited	1.67	1.27	0.25	-0.98	0.33
31. Awe, amazement	1.72	1.46	0.24	-1.31	0.34
31a. Understanding of others' feelings	1.89	1.37	-0.02	-1.2	0.38
32. Safe	1.79	1.21	0.18	-0.91	0.45
33. Feel presence of numinous force, higher power, God	1.49	1.47	0.43	-1.26	0.3
34. Change in feelings about sounds in the room	1.66	1.42	0.26	-1.28	0.41
35. Happy	2.35	1.21	-0.35	-0.59	0.47
36. Sad	1.02	1.34	1.08	-0.07	0.2
36a. Loving	1.86	1.37	-0.06	-1.28	0.37
37. Euphoria	1.58	1.33	0.35	-0.99	0.32
38. Despair	0.6	1.03	1.84	2.6	0.15

39. Feel like crying	0.92	1.29	1.26	0.46	0.18
40. Change in feelings of closeness to people in the room	1.69	1.36	0.12	-1.17	0.34
41. Change in "amount" of emotions	1.93	1.3	0.03	-0.95	0.39
42. Emotions seem different than usual	1.7	1.28	0.17	-0.99	0.34
43. Feeling of oneness with the universe	1.66	1.43	0.23	-1.32	0.33
44. Feel isolated from people and things	0.76	1.08	1.47	1.53	0.15
45. Feel reborn	0.82	1.16	1.25	0.41	0.21
46. Satisfaction with the experience	2.73	1.15	-0.73	-0.16	0.55
47. Like the experience	2.67	1.15	-0.73	-0.12	0.53
48. How soon would you like to repeat the experience?	2.11	1.28	0.07	-0.88	0.42
49. Is this an experience you would like to have regularly?	1.66	1.3	0.24	-0.99	0.33
50. An odor	0.54	0.95	1.79	2.43	0.14
51. A taste	0.55	0.91	1.79	2.65	0.14
52. A sound or sounds accompanying the experience	1.1	1.34	0.83	-0.69	0.27
53. Sense of silence or deep quiet	1.45	1.33	0.51	-0.87	0.29
54. Sounds in room sound different	1.53	1.32	0.31	-1.15	0.31
55. Difference in distinctiveness of sounds	1.68	1.33	0.11	-1.3	0.42
56. Auditory synesthesia	0.85	1.19	1.34	0.81	0.17
57. Visual effects	1.98	1.42	-0.03	-1.21	0.4
58. Room looked different	1.51	1.33	0.45	-0.88	0.3
59. Change in brightness of colors / objects in the room	1.46	1.32	0.45	-0.94	0.29
60. Change in acuity of vision / visual distinctiveness of objects	1.44	1.27	0.5	-0.73	0.29
61. Visual field overlaid by patterns	1	1.25	1.07	0.18	0.2
62. Vibration, jiggling or other motion of the visual field	1.26	1.28	0.74	-0.48	0.25
63. Visual synesthesia	0.83	1.18	1.32	0.59	0.21
64. Visual images, visions, or hallucinations	1.74	1.51	0.22	-1.38	0.35
65. Kaleidoscopic nature of images/visions/hallucinations	1.57	1.47	0.43	-1.1	0.31
66. Difference in brightness of visions	1.61	1.35	0.34	-1	0.32
67. Dimensionality of images/visions/hallucinations	1.85	1.37	0.08	-1.14	0.37
68. Movement within visions/hallucinations	1.84	1.42	0.13	-1.17	0.37
69. White light	0.88	1.16	1.15	0.29	0.18
70. Dead or dying	0.48	1.01	2.25	4.12	0.12
71. Sense of speed	1.01	1.24	0.96	-0.24	0.2
72. Deju vu	0.72	1.11	1.49	1.2	0.18
73. Jamais vu	0.57	1.02	1.92	2.9	0.14
74. Contradictory feelings at the same time	1.15	1.29	0.78	-0.6	0.23
75. Sense of chaos	1	1.24	1.09	0.15	0.2
76. Change in strength of sense of self	1.63	1.4	0.28	-1.16	0.33
77. New thoughts or insights	1.83	1.38	0.06	-1.29	0.46
78. Memories of childhood	0.89	1.17	1.16	0.29	0.22
79. Feel like a child	0.98	1.25	1.12	0.14	0.2
80. Change in rate of thinking	1.92	1.31	-0.01	-1.09	0.38
81. Change in quality of thinking	1.92	1.29	0.01	-1.06	0.38
82. Difference in feeling of reality	2.11	1.38	-0.14	-1.09	0.42
83. Dreamlike nature of the experience	1.5	1.3	0.49	-0.86	0.3
84. Thoughts of present or recent past	1.64	1.42	0.26	-1.23	0.33
85. Insights into personal or occupational concerns	1.56	1.39	0.29	-1.26	0.31
86. Change in rate of time passing	1.97	1.33	-0.04	-1.11	0.39

87. Unconscious	0.4	0.81	2.35	5.92	0.1
88. How sane did you feel?	0.9	1.18	1.13	0.27	0.18
89. Urge to close your eyes	1.47	1.29	0.41	-0.93	0.29
90. Change in effort of breathing	0.87	1.07	1.08	0.28	0.22
91. Able to follow the sequence of events	1.87	1.1	0.09	-0.65	0.47
92. Able to "let go"	1.91	1.16	0.04	-0.79	0.48
93. Able to focus attention	1.95	1.07	-0.15	-0.61	0.49
94. In control	1.93	1.06	-0.01	-0.63	0.48
95. Able to move around if asked to do so	1.8	1.2	0.14	-0.93	0.45
96. Awareness of external situation	1.7	1.46	0.25	-1.42	0.43
97. Waxing and waning of the experience	1.98	1.26	-0.14	-0.93	0.4
98. Intensity	2.64	1.18	-0.62	-0.28	0.53
99. High	2.35	1.26	-0.38	-0.7	0.47

Table S2. Factor-level data for each factor from three proposed models of the HRS, including Cronbach's α and McDonald's Ω as measures of internal consistency.

	Mean	Skew	Kurtosis	Difficulty	Discrimination	Ω	α	Variance explained
**New factors**								
Vision	1.70	0.27	-0.82	0.34	0.64	0.97	0.96	0.13
Meaningfulness	1.52	0.42	-0.79	0.31	0.62	0.95	0.94	0.10
Dysphoria	0.69	1.70	2.58	0.15	0.60	0.90	0.88	0.06
Euphoria	1.65	0.30	-0.72	0.33	0.59	0.89	0.86	0.05
Somaesthesia	0.98	1.04	0.51	0.21	0.52	0.87	0.84	0.04
Auditory and Minor Senses	1.25	0.71	-0.34	0.29	0.60	0.90	0.87	0.04
Liking	2.36	-0.35	-0.51	0.47	0.60	0.91	0.84	0.04
Volition	1.85	0.08	-0.85	0.46	0.50	0.87	0.80	0.03
**Clinical clusters (Strassman et al., 1994)**								
Intensity	2.32	-0.38	-0.64	0.47	0.61	0.86	0.85	
Somaesthesia	1.31	0.62	-0.37	0.27	0.48	0.90	0.87	
Affect	1.56	0.44	-0.44	0.32	0.43	0.90	0.85	
Perception	1.44	0.50	-0.75	0.30	0.62	0.96	0.94	
Cognition	1.54	0.41	-0.78	0.32	0.60	0.94	0.92	
Volition	1.69	0.23	-0.71	0.41	0.39	0.82	0.73	
**Bouso et al., 2016**								
Sensitive distortion	1.51	0.38	-0.98	0.32	0.58	0.91	0.87	
Cognitive distortion	1.50	0.43	-0.80	0.30	0.57	0.94	0.92	
Agitation	0.99	0.98	0.23	0.22	0.52	0.86	0.81	
Security / Control	1.69	0.28	-0.44	0.39	0.31	0.82	0.71	
Visual distortion	1.61	0.37	-0.91	0.32	0.74	0.96	0.93	
Quality	1.69	0.28	-0.61	0.35	0.60	0.93	0.90	

*Table S3. Results of ANOVAs examining differences between all drugs and doses for each factor. A significant result signifies that at least one drug and dose combination was significantly different from another. *** p < 0.001.*

Factor	Sum Sq	Mean Sq	NumDF	DenDF	F value	Pr(>F)
Vision	91.844	2.482	37.000	54.833	26.018	<0.001***
Auditory and Minor Senses	21.613	0.584	37.000	50.137	7.574	<0.001***
Somaesthesia	11.819	0.319	37.000	55.982	6.128	<0.001***
Meaningfulness	25.425	0.687	37.000	65.351	11.059	<0.001***
Euphoria	53.109	1.435	37.000	58.898	10.048	<0.001***
Dysphoria	13.888	0.375	37.000	53.075	5.475	<0.001***
Volition	15.282	0.413	37.000	60.053	4.335	<0.001***
Liking	15.628	0.422	37.000	65.577	3.069	<0.001***

*Table S4. Mean weighted factor scores for each drug and dose combination. Two-tailed Wilcoxon tests were used to compare each drug and dose combination to placebo on each factor. The Benjamini-Hochberg procedure was applied to reduce the false discovery rate. ***p <0.001, **p <0.01, *p <0.05.*

Drug and dose	N	Vision	Auditory and Minor Senses	Somaesthesia	Meaningfulness	Euphoria	Dysphoria	Volition	Liking
placebo	*172*	*0.56*	*0.33*	*0.34*	*0.59*	*0.71*	*0.26*	*0.88*	*1.16*
2C-B 20mg	35	**1.15*** **	**0.63*** **	**0.57*** **	**0.78*** **	**1.14*** **	**0.5*** **	0.83	1.3
ayahuasca 113mL	158	**1.09*** **	**0.81*** **	**0.53*** **	**0.98*** **	**0.94*** **	**0.43*** **	**1.26*** **	**1.39*** **
ayahuasca 1ml/kg	39	**0.73*** **	**0.37*** **	**0.31*** **	0.45	0.52	**0.26*** **	0.66	1.22
ayahuasca n.s.	153	**1.1*** **	**0.73*** **	**0.49*** **	**0.9*** **	**1.04*** **	**0.42*** **	0.91	**1.22** **
d-amphetamine 10mg	14	0.49	0.31	0.33	0.35	0.78	0.19	0.84	0.9
d-amphetamine 20mg	14	0.57	0.26	0.32	0.29	0.77	0.13	0.89	1.06
d-Methamphetamine 0.2mg/kg	8	**1.25*** **	**0.78*** **	**0.82*** **	**1.16** **	**1.3** **	**0.72*** **	0.87	1.52
d-Methamphetamine 0.4mg/kg	8	**1.07*** **	**0.76*** **	**0.74*** **	**1.1*** **	**1.13*** **	**0.68*** **	0.96	**1.26***
DMT 0.05mg/kg	20	**0.44** **	0.21	0.14	0.31	0.58	0.06	0.58	**1.47** **
DMT 0.1mg/kg	8	0.66	0.18	0.17	0.36	0.83	0.03	0.4	1.57
DMT 0.2mg/kg	12	**1.47*** **	0.35	0.12	**0.56***	**1.26*** **	**0.28*** **	0.64	**1.76*** **
DMT 0.3mg/kg	8	**1.76*** **	**0.43***	0.14	0.69	**1.28** **	0.14	0.79	**1.89*** **
DMT 0.4mg/kg	15	**1.86*** **	0.42	**0.52*** **	**0.9*** **	**1.15*** **	**0.54*** **	**1.33** **	1.19
ketamine 0.5mg/kg	5	**0.28** **	**0.12** **	0.32	0.35	**0.69***	**0.11** **	1.43	0.73
mCPP 0.5mg/kg	14	**0.6***	0.39	0.44	0.35	0.68	0.23	1.17	1
mCPP 0.75mg/kg	14	**0.49** **	**0.2***	**0.47** **	0.34	0.86	**0.2***	**0.89***	1.24
MDE 2 mg/kg	16	**0.97*** **	**0.71*** **	**0.71*** **	**0.78*** **	**1.1*** **	**0.66*** **	1.27	**1.15*** **
MDMA 0.25mg/kg	1	0.09	0	0.49	0.58	0.32	0	0.33	1.05
MDMA 0.5mg/kg	4	0.65	0.52	0.35	0.85	1.15	0.16	1.04	1.02
MDMA 0.75mg/kg	4	0.53	0.16	0.31	0.81	0.77	0.26	0.92	1.01
MDMA 1.25mg/kg	4	0.39	0.22	0.36	**0.8***	0.74	0.18	1.06	1.12
MDMA 1.5mg/kg	3	0.48	0.34	0.18	0.72	0.81	0.16	1.26	1.15
MDMA 1.75mg/kg	4	0.19	0.06	0.22	0.68	0.84	0.03	**1.46***	0.97
MDMA 1mg/kg	18	**0.71***	0.36	**0.56*** **	0.47	**1.1*** **	0.21	0.98	1.37

MDMA 2.25mg/kg	5	1.01	0.4	**0.37***	**1.14****	**1.1***	0.3	**0.51***	1.23
MDMA 2.5mg/kg	1	1.74	0.88	0.85	1.94	1.49	0.72	0.38	1.54
MDMA 2mg/kg	17	**0.44****	**0.26***	**0.24*****	0.39	**0.65*****	0.16	1.01	**1.06****
MDMA n.s.	1	0.57	0.14	0.24	1.06	1.61	0	1.89	1.97
methylphenidate 40mg/70kg	30	**0.48*****	0.2	**0.3*****	**0.52*****	**0.61*****	0.12	0.83	1.03
psilocybin 0.025mg/kg	7	0.56	0.36	0.41	0.75	0.76	0.18	1.34	1.27
psilocybin 0.1mg/kg	9	**0.7****	**0.4*****	0.38	**0.73***	**0.91***	0.38	1.07	1.37
psilocybin 0.2mg/kg	22	**1.12*****	**0.66*****	**0.7*****	**1.07*****	**1.28*****	**0.61*****	1.17	**1.44****
psilocybin 0.3mg/kg	34	**1.01*****	**0.56*****	**0.39*****	**1.04*****	**1.02*****	**0.29*****	**1.37*****	**1.45***
psilocybin 0.43mg/kg	30	**1.23*****	**0.53*****	**0.56*****	**0.98*****	**1.32*****	**0.49*****	0.88	**1.16****
salvinorin A 1017ug	30	**1.24*****	**0.75*****	**0.38***	**0.74*****	**1.03*****	**0.38*****	**1.06****	1.25
THC 15mg	27	**0.57*****	**0.42****	**0.33*****	0.58	**0.71****	**0.24****	**1.06***	1.02
THC 7.5mg	27	**0.38***	0.27	**0.28***	0.4	**0.51***	**0.12***	**0.82***	**0.92***

*Table S5. Results of ANOVAs examining differences between five drug classes and placebo for each factor. A significant result signifies that at least one drug class was significantly different from another. *** p < 0.001.*

**Factor**	**Sum Sq**	**Mean Sq**	**NumDF**	**DenDF**	**F value**	**Pr(>F)**
Vision	70.34	14.07	5.00	509.19	106.79	<0.001***
Auditory and Minor Senses	13.78	2.76	5.00	358.85	30.85	<0.001***
Somaesthesia	8.37	1.67	5.00	364.69	28.71	<0.001***
Meaningfulness	16.16	3.23	5.00	402.17	42.20	<0.001***
Euphoria	35.98	7.20	5.00	470.35	44.05	<0.001***
Dysphoria	10.01	2.00	5.00	284.38	25.92	<0.001***
Volition	6.62	1.32	5.00	520.98	13.72	<0.001***
Liking	4.10	0.82	5.00	370.84	5.62	<0.001***

*Table S6. Results of pairwise Wilcoxon tests contrasting psychedelics with each other drug class and placebo. Benjamini-Hochberg correction was applied to reduce the false discovery rate. *** p < 0.001, ** p < 0.01, * p < 0.05.*

	**Psychedelics**	**Dissociatives**	**Empathogens**	**THC**	**Stimulants**	**Placebo**
Vision	1.18	1.24	**0.69****	**0.46****	**0.36****	**0.26****
Auditory and Minor Senses	0.72	0.69	**0.42****	**0.22****	**0.14****	**0.14****
Somaesthesia	0.51	**0.29****	0.58	**0.4***	**0.28****	**0.17****
Meaningfulness	0.93	**0.66****	**0.72****	**0.4****	**0.51****	**0.35****
Euphoria	1.09	0.94	1.12	**0.63****	**0.65****	**0.41****
Dysphoria	0.44	0.37	**0.28****	**0.22****	**0.08****	**0.11****
Volition	1.00	**1.15***	1.11	**1.13***	**0.72****	**0.86****
Liking	1.34	1.22	**1.43***	**0.83****	**1.16****	**1.08****
N	543	35	68	54	58	172

*Table S7. Results of pairwise Wilcoxon tests contrasting psychedelics with each other drug class and placebo for each item on the Meaningfulness factor. Benjamini-Hochberg correction was applied to reduce the false discovery rate. *** p < 0.001, ** p < 0.01, * p < 0.05.*

Item on "Meaningfulness" factor	Psychedelics	Dissociatives	Empathogens	THC	Stimulants	Placebo
27a. Self-accepting	2.34	2.26	2.56	**1.24****	2.09	**1.45****
27b. Forgiving yourself or others	2.01	**1.37***	2.18	**0.85****	**1.53***	**1.05****
31a. Understanding of others' feelings	2.07	**1.09****	2.12	**0.89****	**1.21****	**0.85****
33. Feel presence of numinous force, higher power, God	1.72	1.66	**1.01****	**0.48****	**1.05****	**0.61****
36. Sad	1.26	**0.31****	**0.47****	**0.33****	1.24	**0.35****
36a. Loving	1.93	1.46	2.29	**1.28****	**1.05****	**1.14****
39. Feel like crying	1.15	**0.66***	**0.38****	**0.15****	0.79	**0.34****
40. Change in feelings of closeness to people in the room	1.93	**1.09****	1.79	**0.52****	**0.88****	**0.62****
41. Change in "amount" of emotions	2.16	1.74	1.90	**0.85****	**1.00****	**0.6****
43. Feeling of oneness with the universe	1.94	1.46	**1.29****	**0.43****	**0.83****	**0.59****
45. Feel reborn	0.95	0.69	0.74	**0.24****	**0.34****	**0.38****
53. Sense of silence or deep quiet	1.60	1.26	1.41	**0.74****	1.26	**1.04****
**74. Contradictory feelings at the same time**	1.34	**0.66****	**0.75****	**0.65****	**0.43****	**0.3****
76. Change in strength of sense of self	1.87	1.43	**1.31****	**0.8****	**0.71****	**0.42****
77. New thoughts or insights	2.11	1.69	**1.24****	**0.85****	**1.12****	**0.52****
**78. Memories of childhood**	1.03	**0.49***	**0.59***	**0.61***	**0.67***	**0.25****
79. Feel like a child	1.13	1.03	**0.72***	**0.41****	**0.4****	**0.28****
81. Change in quality of thinking	2.14	1.86	**1.4****	**1.41****	**0.62****	**0.66****
**84. Thoughts of present or recent past**	1.88	**1.00****	**1.31****	**0.87****	**1.05****	**0.95****
**85. Insights into personal or occupational concerns**	1.77	**1.11***	**1.28***	**1.00***	**1.16****	**0.6****
N	543	35	68	54	74	172
