# Supplementary material for: The Hallucinogen Rating Scale: Updated Factor Structure in a Large, Multistudy Sample
Source: Biol Psychiatry Glob Open Sci. 2024 Dec 19;5(2):100436. doi: 10.1016/j.bpsgos.2024.100436 (PMC11804565; doi:10.1016/j.bpsgos.2024.100436)
Supplement: Hallucinogen Rating Scale Version 4 English [file mmc2.pdf]

HRS (Version 4)

Beginning on the next page is a list of statements referring to effects of the drug you received. For each statement, please mark the answer that corresponds to the **most intensely** you experienced that effect during your session.

Please mark **only one** answer for each item; mark the one that seems best, even if none matches your experience exactly. For example, "Visual effects," if you experienced extremely intense visual effects during the time period specified, mark "Extremely." Do not worry if your answers to some questions are opposite to others. If two opposite experiences occurred at some point during the time period, answer each one according to what you experienced.

Name ________________ Date ________ Dose _____ Protocol _____ (Session _____)

Copyright© 1994-2024, by Rick J. Strassman, M.D., Department of Psychiatry, University of New Mexico, School of Medicine, Albuquerque, NM. All rights reserved.

	Statement		Not applicable, no effect
1	Amount of time between when the drug was administered and feeling an effect	__________ seconds / minutes (circle one)	

	Statement	Not at all	Slightly	Moderately	Very much	Extremely
2	A "rush"					
2a	Where was this rush located in your body?					
3	Change in salivation					
3a	Was your mouth drier, wetter, or both? (circle one)					
4	Body feels different					
4a	Please describe					
5	Changes in sense of bodyweight					
5a	Was your body lighter, heavier, or both? (circle one)					
6	Feel as if moving/falling/flying through space					
7	Change in body temperature					
7a	Did you feel warmer, cooler, or both? (circle one)					
8	Electric/tingling feeling					
9	Pressure or weight in chest or abdomen					
9a	Physically loose, limber or flexible					
10	Shaky feelings inside					
11	Body shake/tremble on the outside					
12	Feel heart beating					
13	Heart skipping beats					
14	Nausea					
15	Physically comfortable					
16	Physically restless					
17	Flushed					
18	Urge to urinate					
19	Urge to move bowels					
20	Sexual feelings					
21	Feel removed, detached, separated from body/ did you lose awareness of your body?					
22	Change in skin's sensitivity					
22a	Was your skin more sensitive, less sensitive, or both? (circle one)					
23	Sweating					

	Statement	Not at all	Slightly	Moderately	Very much	Extremely
24	Headache					
25	Anxious					
26	Frightened					
27	Panic					
27a	Self-accepting					
27b	Forgiving yourself or others					
28	At ease					
29	Feel like laughing					
30	Excited					
31	Awe, amazement					
31a	Understanding of others' feelings					
32	Safe					
33	Perceiving the presence of an other being					
33a	What kind of other being did you perceive?<br>Please describe it briefly.					
33b	What was the attitude of this other being toward you?<br>For example, did it seem benevolent, hostile, or uninterested?					
33c	Did you interact with this other being?					
33d	Please briefly describe how you interacted with it.					
34	Change in feelings about sounds around you					
34a	Did you find the sounds around you more pleasant, less pleasant, or both? (circle one)					
35	Happy					
36	Sad					
36a	Loving					
37	Euphoria					
38	Despair					
39	Feel like crying					
40	Change in feelings of closeness to people who were with you					
40a	Did you feel less close to them, more close, or both? (circle one)					

	Statement	Not at all	Slightly	Moderately	Very much	Extremely
41	Change in "amount" of emotions					
41a	Were you less emotional, more emotional, or both? (circle one)					
42	Emotions seem different than usual					
43	Feeling of oneness with the universe					
44	Feel isolated from people and things					
45	Feel reborn					
46	Satisfaction with the experience					
47	Like the experience					

	Statement	Never again	Someday, but not in the next year	Within a year	Within a month	Within a week	As soon as possible
48	How soon would you like to repeat the experience?						

	Statement	Not at all	Slightly	Moderately	Very much	Extremely
49	Is this an experience you would like to have regularly?					
50	An odor					
51	A taste					
52	Hearing a sound or voice within the experience					
53	Sense of silence or deep quiet					
54	Sounds in room sound different					
55	Difference in distinctiveness of sounds					
55a	Less distinct, more distinct, or both? (circle one)					
56	Auditory synesthesia					
57	Visual effects					
58	Room looked different					
59	Change in brightness of colors / objects					
59a	Were objects brighter, duller, or both? (circle one)					
60	Change in acuity of vision / visual distinctiveness of objects					
60a	Were objects sharper, blurrier, or both? (circle one)					
61	Visual field overlaid by patterns					
62	Vibration, jiggling or other motion of the visual field					
63	Visual synesthesia					
64	Visual images, visions, or hallucinations					
65	Kaleidoscopic nature of images/visions/hallucinations					

	Statement	Not at all	Slightly	Moderately	Very much	Extremely
66	Difference in brightness of visions compared to usual daylight vision					
66a	Brighter, duller, or both? (circle one)					
66b	What were the predominant colors?					

	Statement	Not applicable, none seen	Linear (one-dimensional)	Flat/planer (two-dimensional)	Three-dimensional	Multi-dimensional	Beyond dimensionality
67	Dimensionality of images/visions/hallucinations						

	Statement	Not at all	Slightly	Moderately	Very much	Extremely
68	Movement within visions/hallucinations					
69	White light					
70	Dead or dying					
71	Sense of speed					
72	Deja vu					
73	Jamais vu					
74	Contradictory feelings at the same time					
75	Sense of chaos					
76	Change in strength of sense of self					
76a	Was your sense of self stronger, weaker, or both? (circle one)					
77	New thoughts or insights					
78	Memories of childhood					
79	Feel like a child					
80	Change in rate of thinking					
80a	Was your thinking faster, slower, or both? (circle one)					
81	Change in quality of thinking					
81a	Was your thinking sharper, duller, or both? (circle one)					
82	Difference in feeling of reality of experiences compared to everyday experience					
82a	Did the experience seem more real, less real, or both? (circle one)					
83	Dreamlike nature of the experiences					
84	Thoughts of present or recent past					
85	Insights into personal or occupational concerns					
86	Change in rate of time passing					
86a	Was time passing faster, slower, or both? (circle one)					

	Statement	Not at all	Slightly	Moderately	Very much	Extremely
87	Unconscious					
88	Change in sense of sanity					
88a	Did you feel more sane, less sane, or both? (circle one)					
89	Urge to close your eyes					
90	Change in effort of breathing					
90a	Was your breathing more relaxed, more difficult, or both? (circle one)					
91	Able to follow the sequence of events					
92	Able to "let go"					
93	Able to focus attention					
94	In control					
95	Able to move around if asked to do so					
96	Could you remind yourself of where you were, that you have taken a drug, and that the experience would be over at some point?					
97	Waxing and waning of the experience					
98	Intensity					
99	High					
100	Dose you think you received					
101	I had no thoughts					
102	Feeling peaceful					
103	Sense of experiencing “more” than everyday experience					
104	Inanimate objects seemed alive					
105	Feeling exhausted					
106	Feeling stimulated					
107	Sense of meaningfulness					
108	Experience of beauty					
109	Feeling free					
110	Feeling confused or disoriented					
Any other comments?						

HRS (Version 4) – Scoring Instructions

Below are instructions for scoring the HRS based on the 8-factor model (Calder et al., 2024). Instructions for scoring the original clinical clusters (Strassman et al., 1994) are also included for completeness.

1. Data entry and reverse scoring

Most numerical questions are entered as "0" for "Not at all," and "4" for "Extremely." There are exceptions:

- #48: "Never again" is scored "0," while "As soon as possible" is scored "4."
- #67: “Not applicable” is scored as a missing value. "Linear" is scored as "0," "Flat/planar" is scored as "1,"and so on up to "Beyond dimensionality" as "4.”

The following items are **reverse scored**: 32, 91, 92, 93, 94, 95, 96

Note: There are several questions which we’ve not yet placed into factors. There are for possible inclusion in future versions of the HRS. Please enter them nevertheless.

2. Factor scores (8-factor model)

Factors scores are calculated using the mean score of all items within that factor.

**Factor**	**Items**
Vision	2, 4-6, 21, 57-62, 64-68, 71, 80, 82, 83, 86, 98, 99
Meaningfulness	27a, 27b, 31a, 33, 36, 36a, 39-41, 43, 45, 53, 74, 76-79, 81, 84, 85
Euphoria	20, 29-31, 35, 37, 42
Dysphoria	25-27, 38, 44, 70, 75, 87, 88
Auditory and Minor Senses	22, 34, 50, 52, 54, 55, 63
Liking	15, 28, 46-49
Somaesthesia	3, 8-13, 16, 90
Volition	32, 91-96

3. Clinical clusters (optional)

Scores for the original six clinical clusters are calculated using the mean score of all items within each cluster:

**Cluster**	**Items**
Intensity	97-99
Somaesthesia	2-11, 16, 20, 21
Affect	25, 26, 29-33, 37, 40-45, 47-49
Perception	17, 22, 52, 54, 55, 57-62, 64-69
Cognition	71, 74, 75-77, 80-83, 85, 86, 88
Volition	89-96

Literature

Calder AE, Qualls CR, Hasler G, et al. (2024) The Hallucinogen Rating Scale: Updated factor structure in a large, multi-study sample.

Strassman RJ, Qualls CR, Uhlenhuth EH, et al. (1994) Dose-response study of N,N-dimethyltryptamine in humans. II. Subjective effects and preliminary results of a new rating scale. *Arch Gen Psychiatry* 51(2): 98-108.
