# Supplementary material for: The Hallucinogen Rating Scale: Updated Factor Structure in a Large, Multistudy Sample
Source: Biol Psychiatry Glob Open Sci. 2024 Dec 19;5(2):100436. doi: 10.1016/j.bpsgos.2024.100436 (PMC11804565; doi:10.1016/j.bpsgos.2024.100436)
Supplement: Hallucinogen Rating Scale Version 4 German [file mmc3.pdf]

HRS (Version 4)

Beginnend auf der nächsten Seite finden Sie eine Liste mit Aussagen. Diese beziehen sich auf die Effekte der Substanz, welche Sie erhalten haben. Bitte wählen Sie jeweils die Antwortoption, die Ihrer **intensivsten** Erfahrung dieses Effekts während Ihrer Sitzung entspricht.

Bitte wählen Sie **nur eine** Antwortoption pro Frage. Wählen Sie diejenige, die am besten scheint, auch wenn keine Antwort exakt auf Ihre Erfahrung zutrifft. Wenn Sie zum Beispiel extrem intensive visuelle Effekte in dem angegeben Zeitraum erlebt haben, wählen Sie "extrem" bei der Kategorie "visuelle Effekte". Machen Sie sich keine Sorgen, falls Ihre Antworten auf einige Fragen gegensätzlich zu anderen Antworten sind. Falls zu einem Zeitpunkt innerhalb des Zeitraums zwei gegenteilige Erfahrungen stattgefunden haben, beurteilen Sie beide davon, entsprechend Ihrer Erfahrungen.

Name ____________________ Datum __________ Dosis ______ Protokoll ______ (Sitzung) ______

Copyright© 1994-2024, Dr. med. Rick J. Strassman, Departement für Psychiatrie, Universität New Mexiko, Medizinische Fakultät, Albuquerque, NM. Deutsche Übersetzung von David Elmiger, unterstützt von Adrian Hase, Vincent Diehl, Morten Lietz, Abigail Calder und Prof. Dr. med. Gregor Hasler. Alle Rechte vorbehalten.

	Aussage		Nicht anwendbar, kein Effekt
1	Zeitspanne zwischen Verabreichung der Substanz und dem Spüren eines Effekts	__________ Sekunden / Minuten (umkreisen Sie eines)	

	Aussage	Überhaupt nicht	Leicht	Moderat	Sehr viel	Extrem
2	Ein "Rausch" von Energie					
2a	Wo befand sich dieser "Rausch" in Ihrem Körper?					
3	Veränderung des Speichelflusses					
3a	War Ihr Mund trockener, feuchter oder beides? (umkreisen Sie eines)					
4	Körper fühlt sich anders an					
4a	Bitte beschreiben					
5	Veränderte Wahrnehmung des Körpergewichts					
5a	War Ihr Körper leichter, schwerer oder beides? (umkreisen Sie eines)					
6	Sich fühlen, als ob man durch den Raum bewegen/fallen/fliegen würde					
7	Veränderung der Körpertemperatur					
7a	Fühlten Sie sich wärmer, kälter oder beides? (umkreisen Sie eines)					
8	Elektrisches/kribbelndes Gefühl					
9	Druck oder Gewicht in der Brust oder im Bauch					
9a	Körperlich locker, geschmeidig oder flexibel					
10	Sich innerlich zittrig fühlen					
11	Körperschütteln/-Zittern					
12	Den Herzschlag spüren					
13	Das Herz setzt Schläge aus					
14	Übelkeit					
15	Körperlich behaglich					
16	Körperlich ruhelos					
17	Errötet					
18	Harndrang					
19	Drang zum Stuhlgang					
20	Sexuelle Gefühle					
21	Sich vom Körper entfernt, losgelöst, oder getrennt fühlen/haben Sie das Bewusstsein für Ihren Körper verloren?					
22	Veränderung in Empfindlichkeit der Haut					
22a	War Ihre Haut empfindlicher, weniger empfindlich oder beides? (umkreisen Sie eines)					

	Aussage	Überhaupt nicht	Leicht	Moderat	Sehr viel	Extrem
23	Schwitzen					
24	Kopfschmerzen					
25	Ängstlich					
26	Erschrocken					
27	Panisch					
27a	Selbstakzeptierend					
27b	Sich selbst oder anderen vergeben					
28	Unbesorgt					
29	Lust zu lachen					
30	Aufgeregt					
31	Ehrfurcht, Erstaunen					
31a	Verständnis der Gefühle anderer					
32	Sicher					
33	Die Präsenz eines "anderen Wesen" wahrnehmen					
33a	Was für ein "anderes Wesen" haben Sie wahrgenommen? Bitte beschreiben Sie es kurz.					
33b	Was war die Haltung dieses "anderen Wesens" Ihnen gegenüber? Zum Beispiel schien es wohlwollend, feindlich oder uninteressiert?					
33c	Haben Sie mit diesem anderen Wesen interagiert?					
33d	Bitte beschreiben Sie kurz, wie Sie mit ihm interagiert haben.					
34	Veränderung der Gefühle bezüglich Geräuschen um Sie herum					
34a	Fanden Sie die Geräusche um Sie herum angenehmer, weniger angenehm oder beides? (umkreisen Sie eines)					
35	Glücklich					
36	Traurig					
36a	Liebend					
37	Euphorie					
38	Verzweiflung					
39	Das Bedürfnis zu weinen verspüren					
40	Veränderung des Gefühls der Nähe zu Menschen die bei Ihnen waren					
40a	Fühlten Sie sich ihnen weniger nah, näher oder beides? (umkreisen Sie eines)					

	Aussage	Überhaupt nicht	Leicht	Moderat	Sehr viel	Extrem
41	Veränderung der "Menge" an Emotionen					
41a	Waren Sie weniger emotional, emotionaler oder beides? (umkreisen Sie eines)					
42	Emotionen scheinen anders als sonst					
43	Gefühl des Einssein mit dem Universum					
44	Sich von Menschen und Dingen isoliert fühlen					
45	Sich wiedergeboren fühlen					
46	Zufriedenheit mit der Erfahrung					
47	Die Erfahrung mögen					

	Aussage	Nie wieder	Eines Tages, aber nicht im nächsten Jahr	Innerhalb eines Jahres	Innerhalb eines Monats	Innerhalb einer Woche	So bald wie möglich
48	Wie bald möchten Sie die Erfahrung wiederholen?						

	Aussage	Überhaupt nicht	Leicht	Moderat	Sehr viel	Extrem
49	Ist dies eine Erfahrung, die Sie regelmässig machen möchten?					
50	Ein Geruch					
51	Ein Geschmack					
52	Ein Geräusch oder eine Stimme während der Erfahrung hören					
53	Ein Gefühl der Stille oder tiefer Ruhe					
54	Geräusche im Raum klingen anders					
55	Unterschied in der Unterscheidbarkeit der Geräusche					
55a	Einfacher zu unterscheiden, schwieriger zu unterscheiden oder beides? (umkreisen Sie eines)					
56	Auditorische Synästhesie					
57	Visuelle Effekte					
58	Der Raum sah anders aus					
59	Veränderung in der Helligkeit von Farben/Objekten					
59a	Waren Objekte heller, matter oder beides? (umkreisen Sie eines)					
60	Veränderung der Sehschärfe / visuelle Unterscheidbarkeit von Objekten					
60a	Waren Objekte schärfer, verschwommener oder beides? (umkreisen Sie eines)					
61	Sichtfeld von Mustern überlagert					
62	Vibration, Wackeln oder andere Bewegung im Sichtfeld					
63	Visuelle Synästhesie					
64	Visuelle Bilder, Visionen oder Halluzinationen					
65	Kaleidoskopische Natur von Bildern/Visionen/Halluzinationen					

	Aussage	Überhaupt nicht	Leicht	Moderat	Sehr viel	Extrem
66	Unterschied in der Helligkeit von Visionen verglichen mit der üblichen Sicht bei Tageslicht					
66a	Heller, matter, oder beides? (umkreisen Sie eines)					
66b	Was waren die vorherrschenden Farben?					

	Aussage	Nicht anwendbar, keine gesehen	Linear (ein-dimensional)	Flach/eben (zwei-dimensional)	Drei-dimensional	Mehr-dimensional	Jenseits der Dimensionalität
67	Dimensionalität von Bildern/Visionen/Halluzinationen						

	Aussage	Überhaupt nicht	Leicht	Moderat	Sehr viel	Extrem
68	Bewegung innerhalb von Visionen/Halluzinationen					
69	Weisses Licht					
70	Tot oder sterben					
71	Gefühl von Geschwindigkeit					
72	Déjà-vu-Erlebnis					
73	Jamais-vu-Erlebnis					
74	Widersprüchliche Gefühle zur gleichen Zeit					
75	Gefühl von Chaos					
76	Veränderung der Stärke des Selbstgefühls					
76a	War Ihr Selbstgefühl stärker, schwächer, oder beides? (umkreisen Sie eines)					
77	Neue Gedanken oder Einsichten					
78	Kindheitserinnerungen					
79	Sich wie ein Kind fühlen					
80	Veränderung in Geschwindigkeit der Gedanken					
80a	War Ihr Denken schneller, langsamer oder beides? (umkreisen Sie eines)					
81	Veränderung in Qualität des Denkens					
81a	War Ihr Denken schärfer, stumpfer, oder beides? (umkreisen Sie eines)					
82	Unterschied im Gefühl der Realität der Erfahrungen verglichen mit der Alltagserfahrung					
82a	Schien die Erfahrung realer, weniger real oder beides? (umkreisen Sie eines)					
83	Traumartige Natur der Erfahrungen					
84	Gedanken an Gegenwart oder kürzliche Vergangenheit					
85	Einsichten über persönliche oder berufliche Belange					
86	Veränderung des Zeitgefühls					
86a	Verging die Zeit schneller, langsamer, oder beides? (umkreisen Sie eines)					

	Aussage	Überhaupt nicht	Leicht	Moderat	Sehr viel	Extrem
87	Bewusstlos					
88	Veränderung des Gefühls der Vernunft					
88a	Fühlten Sie sich mehr bei Verstand, weniger bei Verstand oder beides? (umkreisen Sie eines)					
89	Drang, Ihre Augen zu schliessen					
90	Veränderung im Aufwand beim Atmen					
90a	War Ihr Atmen entspannter, schwieriger oder beides? (umkreisen Sie eines)					
91	In der Lage, dem Ablauf der Ereignisse zu folgen					
92	In der Lage, "loszulassen"					
93	In der Lage, Aufmerksamkeit zu fokussieren					
94	Unter Kontrolle					
95	In der Lage sich zu bewegen, falls darum gebeten					
96	Hätten Sie sich bewusst machen können, dass Sie im Krankenhaus waren, dass Sie eine Substanz genommen haben, dass die Erfahrung irgendwann vorbei sein würde?					
97	Zunahme und Abnahme der Erfahrung					
98	Intensität					
99	High					
100	Dosis, die Sie glauben, erhalten zu haben					
101	Körperlich locker, geschmeidig oder flexibel					
102	Sich friedlich fühlen					
103	Gefühl, "mehr" als in alltäglicher Erfahrung zu erleben					
104	Leblose Objekte schienen lebendig					
105	Sich erschöpft fühlen					
106	Sich stimuliert fühlen					
107	Gefühl von Bedeutung					
108	Erfahrung von Schönheit					
109	Sich frei fühlen					
110	Sich verwirrt oder desorientiert fühlen					
Irgendwelche weiteren Kommentare?						

HRS (Version 4) – Auswertung

Unten finden Sie Instruktionen für die Auswertung des HRS nach dem 8-Faktoren Modell (Calder et al., 2024). Instruktionen für die Auswertung der originalen „Clinical Clusters“ (Strassman et al., 1994) sind der Vollständigkeit halber ebenfalls enthalten.

1. Dateneingabe und Umpolierung

Die meisten numerischen Fragen werden mit "0" für "überhaupt nicht" und "4" für "extrem" eingegeben. Es gibt Ausnahmen:

- #48: "Nie wieder" wird als "0" eingegeben, "So bald wie möglich" wird als "4" eingegeben.
- #67: “Nicht anwendbar” wird als fehlender Wert betrachtet. "Linear" wird als "0" eingegeben, "Flach/eben" als "1," und so weiter bis "Jenseits der Dimensionalität" als "4.”

Die folgenden Fragen werden **umpoliert**: 32, 91, 92, 93, 94, 95, 96

Hinweis: Es gibt mehrere Fragen, die wir noch nicht in Faktoren unterteilt haben. Sie sind für eine mögliche Aufnahme in zukünftige Versionen des HRS vorgesehen. Bitte geben Sie diese Fragen trotzdem ein.

2. Faktorenwerte (8-Faktoren Modell)

Faktorenwerte werden anhand des Mittelwerts aller Items innerhalb des jeweiligen Faktors berechnet.

**Faktor**	**Items**
Vision	2, 4-6, 21, 57-62, 64-68, 71, 80, 82, 83, 86, 98, 99
Meaningfulness	27a, 27b, 31a, 33, 36, 36a, 39-41, 43, 45, 53, 74, 76-79, 81, 84, 85
Euphoria	20, 29-31, 35, 37, 42
Dysphoria	25-27, 38, 44, 70, 75, 87, 88
Auditory and Minor Senses	22, 34, 50, 52, 54, 55, 63
Liking	15, 28, 46-49
Somaesthesia	3, 8-13, 16, 90
Volition	32, 91-96

3. Clinical clusters (optional)

Die Werte für die ursprünglich „Clinical Clusters“ werden anhand des Mittelwerts aller Items innerhalb jedes Clusters berechnet:

**Cluster**	**Items**
Intensity	97-99
Somaesthesia	2-11, 16, 20, 21
Affect	25, 26, 29-33, 37, 40-45, 47-49
Perception	17, 22, 52, 54, 55, 57-62, 64-69
Cognition	71, 74, 75-77, 80-83, 85, 86, 88
Volition	89-96

Literatur

Calder AE, Qualls CR, Hasler G, et al. (2024) The Hallucinogen Rating Scale: Updated factor structure in a large, multi-study sample.

Strassman RJ, Qualls CR, Uhlenhuth EH, et al. (1994) Dose-response study of N,N-dimethyltryptamine in humans. II. Subjective effects and preliminary results of a new rating scale. *Arch Gen Psychiatry* 51(2): 98-108.
